# Marital Bargaining and Assortative Matching on Fertility Preference : Evidence based on Cross-sectional Data in China

**DOI:** 10.12688/f1000research.151196.1

**Published:** 2024-06-19

**Authors:** Meiyi Zhuang, Hisahiro Naito

**Affiliations:** 1 Graduate School of Humanities and Social Sciences, University of Tsukuba, Tsukuba Tennodai 1-1-1, Ibaraki, 305-8571, Japan; 2 Graduate School of Humanities and Social Sciences, University of Tsukuba, Tsukuba Tennodai 1-1-1, Ibaraki, 305-8571, Japan

**Keywords:** Fertility preference, assortative matching, marriage, bargaining, male-female ratio, China

## Abstract

**Background:**

Despite the relaxation of fertility restrictions, China’s birth rate continues to decline. The Universal Two-Child Policy encourages couples to consider having a second child, often leading to a bargaining process between spouses with differing preferences. Additionally, the skewed sex ratio has increased Chinese women’s bargaining power, highlighting the importance of analyzing fertility decisions through marital bargaining.

**Methods:**

This paper investigates second-child fertility decisions using data from the 2018 China Family Panel Studies and employs Ordinary Least Squares regression. The study examines assortative matching based on fertility preferences and uses the 2020 provincial-level sex ratio for individuals aged 20–39 as a proxy for women’s bargaining power in the marriage market.

**Results:**

The study shows that achieving consensus on having a second child requires cooperation between spouses, particularly when their fertility preferences differ. The study also reveals that marriage matching is not random; individuals are more likely to partner with those who share the same second-child preference. Additionally, women with greater bargaining power positively influence their husbands’ desired family size, a correlation not observed in males.

**Conclusion:**

The study concludes that second-child fertility decisions in China are significantly influenced by marital bargaining and the increased bargaining power of women due to the skewed sex ratio. Cooperation between spouses with differing fertility preferences is crucial for reaching a consensus on having a second child.

## Introduction

Chinese society and economy have undergone significant shifts over the past seven decades. Social issues have evolved from a vast population base with low human capital following the establishment of the People’s Republic of China, to a demographic characterized by an aging population and a labor force shortage in recent years. Correspondingly, fertility policy transitions can be divided into three periods: the first, following 1949, encouraged higher birth rates to support the burgeoning workforce demands; the second, beginning in the 1970s with the “Later, Longer, Fewer” campaign and later reinforced by the One–Child Policy, aimed to control the booming population; and the third, a response to declining fertility rates and an aging population, where a series of two-child policies emerged. In 2011, couples who were both only children were permitted to have a second child under the Double–Single Two–Child Policy; in 2013, the policy expanded to allow couples to have a second child if either spouse was an only child under the Selective Two-Child Policy; and in 2016, the Universal Two-Child Policy was implemented, allowing all couples to have two children.

**Figure 1.**
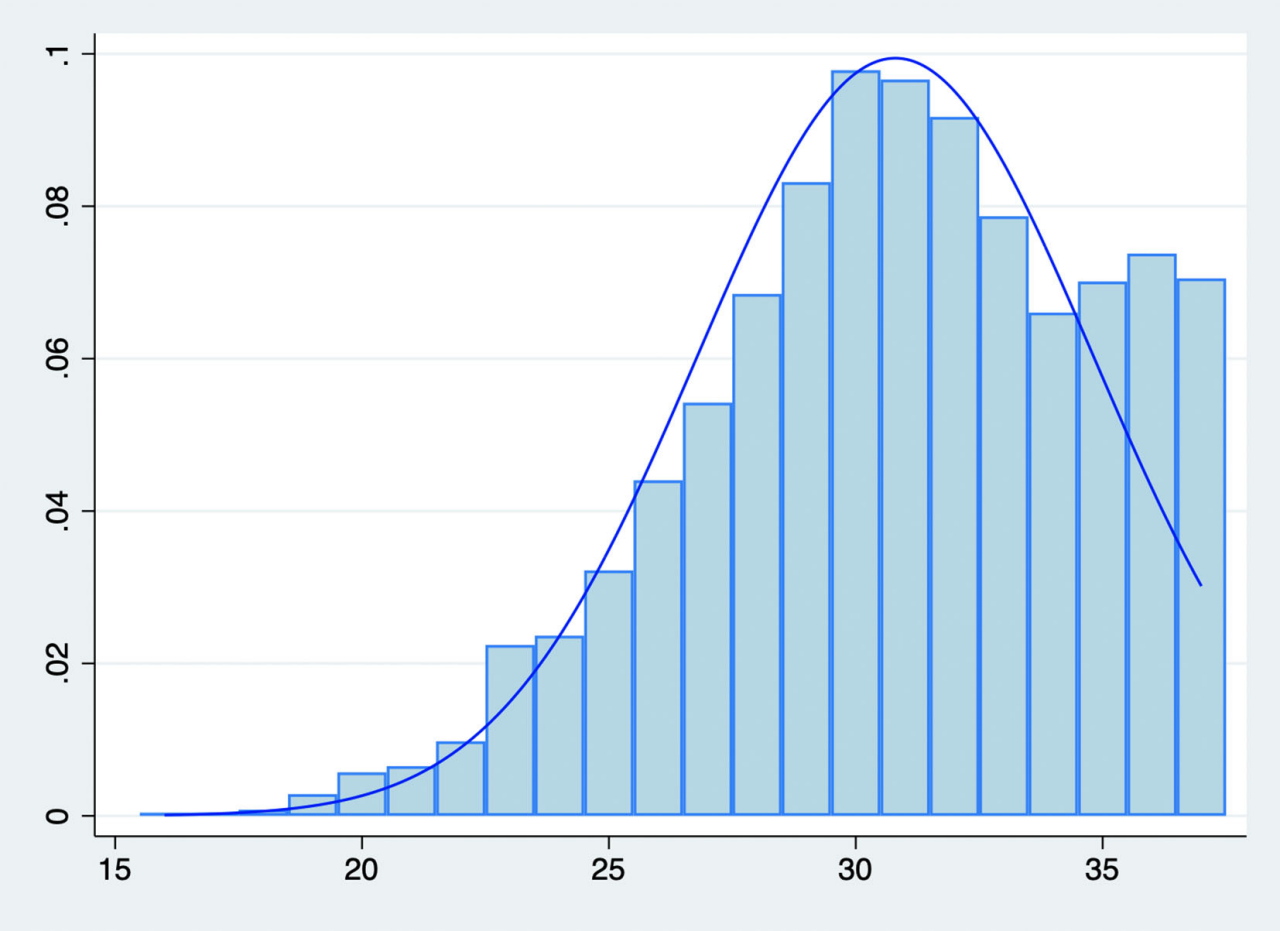
Total Population, Birth, and Natural Growth Rates in China, 2001-2023. Note: The short-dash, dash, and long-dash lines denote the starting times of the Double-Single, Selective, and Universal Two-Child Policies, respectively.

However, the Chinese birth rate has continued to decline in recent years. While there was a temporary increase in birth and natural growth rates following 2016, these rates decreased to new lows in subsequent years, as illustrated in Figure 1. With the belief “more children, more blessings" and the strict enforcement of the One-Child Policy, couples generally do not disagree on having the first child. Disagreements occur over the second and subsequent children, especially when women are empowered by their market status. The Universal Two-Child Policy encourages families to consider a second child, potentially initiating a bargaining process between spouses when their preferences differ. Furthermore, with China’s rapid economic development and a continuously skewed sex ratio, women’s bargaining power within the family has increased ( Bulte et al., 2015). These factors underscore the importance of understanding fertility decisions from the perspective of the marital bargaining process.

Theoretical models highlight the importance of the bargaining process in fertility decisions and illuminate how bargaining over fertility may differ between developed and developing countries. Fertility choices in developing countries are often dominated by a single decision-maker, typically males, who usually prefer a larger family size and possess greater bargaining power. These significant disagreements between spouses about fertility preferences imply that increasing women’s bargaining power could enable them to influence fertility decisions ( Doepke and Tertilt, 2018). In contrast, high-income economies often feature gender-equal labor laws and greater female participation in the labor market, where bargaining over fertility becomes significant because women and men achieve equal bargaining powers ( Doepke et al., 2023). Doepke and Kindermann (2019) propose a veto model for fertility decisions, illustrating that in high-income countries, where men and women achieve equal bargaining power, both spouses have veto power over fertility. This model also considers the spouses’ ability to commit to future consumption. Manser and Brown (1980) integrate marriage decisions within a two-person bargaining framework, allowing husbands and wives with distinct utility functions and preferences to negotiate agreements on crucial aspects such as children, housing, and leisure. These agreements enable them to realize gains from the marriage that would not be possible if they remained single.

The argument above suggests that agreement between spouses on the desired number of children is important. This indicates that in the marriage market, both men and women have strong incentives to seek partners with similar preferences. If a husband and wife share similar views on family size, they may avoid the need for a bargaining process. Therefore, in the presence of heterogeneity in preferences regarding the number of children, assortative matching naturally occurs in the marriage market. Thus, it is crucial to examine the extent to which bargaining and matching based on preferences influence family size.

In this paper, thus, we examine fertility decisions regarding the second child, focusing on differing marital fertility preferences utilizing data from the 2018 China Family Panel Studies survey and applying Ordinary Least Squares (OLS) regression models. We further explore assortative matching based on fertility preferences, using the provincial-level sex ratio as a proxy for women’s bargaining power. To this end, we collected data on the number of males and females aged 20-39 from the 2020 China Population Census Yearbook and calculated the sex ratio by dividing the number of males by the number of females. This approach captures the potential population in the marriage market.

The findings reveal that to have a second child, both the wife and the husband need to cooperate when they have different fertility preferences. Additionally, we found that marriage matching is non-random; specifically, individuals are more likely to pair with partners who share the same preference regarding having a second child. Therefore, we further studied how one spouse’s fertility preference associates with their partner’s. Our conclusions indicate that females with greater bargaining power correlate positively with their husbands’ ideal family size, whereas males do not have a similar correlation.

This paper contributes to the literature on fertility decisions from the perspective of the bargaining process within the Chinese context. Previous research mainly studies fertility decisions from the perspective of females in their reproductive age, which considers the family as a unit that internalizes individual differences to make fertility decisions that maximize household utility. Becker (1960) first applies economic theory to understand fertility decisions in 1960, proposing that children can be regarded as a consumption good that contributes to household utility. The model assumes a unitary household utility function, internalizing differences between family members, where families make rational choices about the number of children based on their preferences, income, and the costs of raising children ( Becker, 1960). Further research considering parental altruism toward children assumes that parental utility also depends on the utility of children in addition to their number, thus household utility includes both the quantity and quality of children. This leads to findings that higher parental altruism leads to higher fertility rates, increased investment in each child's human capital, and greater long-term economic growth due to more capital accumulation, as altruism links the welfare of all generations in a family ( Barro and Becker, 1989; Becker and Barro, 1988). Subsequent research builds on Becker’s work, adapting it to different contexts. For example, Baudin et al. (2015) focus on childlessness in the United States and propose a theory explaining that childlessness among the poor is due to low resources, while among educated women it is due to the high opportunity cost of having children. A study on the Chinese case by Liu and Liu (2020) explores the effectiveness of the Universal Two-Child Policy in China within the same framework, suggesting a threshold for the policy estimated to be lower than two children per household, implying the policy's impact on fertility rates is limited. However, as an essential family decision, fertility is mainly determined by both spouses’ preferences, leading to potential bargaining processes. There is a large amount of empirical literature on other countries based on the perspective of bargaining processes ( Doepke and Kindermann, 2019; Testa et al., 2014). Peng (2020) based on interviews with 53 urban parents in China, reveals that family negotiations significantly shape Chinese reproductive behavior more than individual preferences. However, the quantitative study from the bargaining perspective on the Chinese case is limited.

In addition, this paper contributes to the literature on assortative mating based on fertility preferences. Existing literature primarily examines how assortative mating influences fertility behaviors, with a particular emphasis on educational sorting. For example, Bueno and García-Román (2021) study the Spanish case, indicating that couples with similar educational levels are more likely to have children, especially if both spouses are highly educated. Similarly, a European study finds that educational sorting significantly predicts fertility behaviors; couples with both partners highly educated tend to delay their first child but have more children later, whereas couples with a higher-educated husband and a lower-educated wife are less likely to have a second child ( Nitsche et al., 2018). Tian et al. (2023) also highlight that educational sorting has the most significant impact on women’s fertility preferences compared to age and income sorting using Chinese data. Additionally, the study of marriage matching in China highlights the impact of hukou locality and intergenerational mobility on marital choices. Research from Shanghai indicates that possessing local hukou shapes these choices, enhancing the matching of highly-educated individuals while limiting interactions between hukou residents and less-educated non-hukou migrants ( Qian and Qian, 2017). Urban males are empowered in the marriage market by being granted the same rights as women to pass hukou locality to their children ( Han et al., 2015). Hu (2016) identifies significant associations between the occupational statuses of individuals’ fathers and those of their spouses, as well as with their fathers-in-law. To the best of our knowledge, no existing studies investigate the impact of fertility preferences on marriage matching within the Chinese context. Given these research gaps, this paper aims to provide new insights into how fertility decisions are made through bargaining processes and how fertility preferences could be associated with assortative mating.

The remainder of the paper is organized as follows: Section 2 presents the data and variables used for the empirical analysis. Section 3 outlines the empirical strategy. Section 4 explores how fertility decisions are made based on both spouses’ fertility preferences. Section 5 examines how one spouse’s fertility preference associates with their partner’s preference. Section 6 concludes the paper.

## Methods

### Data set

The primary dataset utilized in this study is the China Family Panel Studies (CFPS), a nationally representative longitudinal sample of Chinese communities, families, and individuals ( Xie and Hu, 2014). The survey includes information on economic activities, educational outcomes, family relationships, migration, and health. Initiated by the Institute of Social Science Survey (ISSS) at Peking University, the baseline survey was conducted in 2010, with follow-up full-sample surveys conducted biennially in 2012, 2014, 2016, 2018, and 2020. Our analysis uses the cross-sectional data from the 2018 CFPS, which includes around 44,000 individuals in 15,000 households from more than 900 counties and cities within 31 provinces, municipalities, and autonomous regions.

The CFPS employs a multi-stage probability sampling strategy to ensure a representative sample of the Chinese population. In the first and second stages, it stratifies and selects provinces and counties using official administrative divisions. Recognizing the geographic disparities inherent in China’s economic development, the design emphasizes geographic representation. In the third stage, households within the chosen counties are selected through a systematic sampling method, targeting 25 households per county to fulfill the study’s requirements.

**Figure 2.**
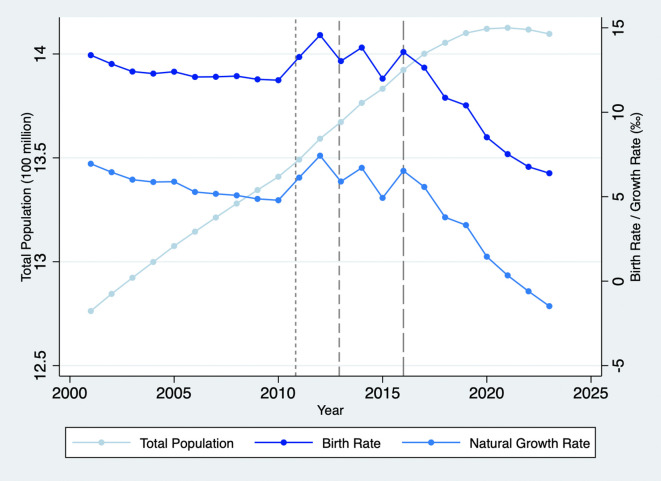
Distribution of Female Age in the Sample, *N*=2,054.

Since we seek to understand the family’s fertility decisions for a second child through the bargaining process, we initially restricted the sample to individuals who responded to questions regarding their ideal number of children. In the 2018 CFPS dataset, 16,268 out of 18,802 females and 16,214 out of 18,552 males provided their responses. Considering both the husband’s and the wife’s preferences, we further constructed a household-level sample that includes information on both spouses. We limited the sample to couples where both spouses responded to the question about the “ideal number of children", as this measures their fertility preference for a second child. We also restricted the age range for women to be equal to or less than 37, corresponding to the possible reproductive age. The distribution of female ages in the sample is shown in Figure 2. Ultimately, the sample comprises 2,054 household observations with detailed information on both husbands and wives. We obtained the permission to use this data set on August 9, 2021.

The main outcome variable in this study is whether the household has two or more children. This variable is derived from the number of children in the family. Since the CFPS does not directly provide complete information on family size, we generated this variable using information available for each child. If both spouses reported their ideal number of children but the actual number of children is missing, we replaced the missing values with 0. In the sample, 46.84% of households have more than two children. The key explanatory variables are the fertility preferences of both spouses for the second child, derived from their reported ideal number of children. In the sample, 79.41% of wives and 80.72% of husbands prefer more than two children. In the second part, the outcome variables are individual fertility preferences, measured by their ideal number of children. On average, both females and males desire fewer than two children. Control variables include both spouses’ employment status (employed or unemployed, without distinguishing between being out of the labor force or unemployed), years of education, age, hukou type (rural or urban), and the family income. We assigned the income to be zero if the individuals are currently not employed. Family income in logarithmic form is calculated by summing the incomes of both spouses and adding one, to avoid issues with missing values when both spouses have no income.

The previous literature has shown, both empirically and theoretically, that a skewed sex ratio increases women’s bargaining power in the marriage market ( Angrist, 2002; Chiappori et al., 2002; Bulte et al., 2015). In this paper, as we are interested in the marital bargaining process regarding family fertility decisions, we use the provincial-level sex ratio as a proxy for bargaining power. We collected data on the number of males and females aged 20-39 from the China Population Census Yearbooks for 2000, 2010, and 2020. The age range of 20-39 captures the potential population in the marriage market. There is no information available for 2018, as the census is conducted every ten years. We calculated the sex ratio by dividing the number of males by the number of females. Given that the statistics are similar across these three census waves, we report only the results using the 2020 sex ratios in this paper. In 2020, the average sex ratio for the population aged between 20 and 39 across 31 provinces was 1.08, implying there are more males than females in the marriage market. The detailed summary statistics for all variables are shown in Table 1.

**Table 1.** Summary statistics.

VARIABLES	Mean	S.d.	Min	Max
**Province**				
2020 sex-ratio (male/female)	1.0797	0.0529	0.9855	1.2086
2010 sex-ratio (male/female)	1.0428	0.0584	0.9536	1.2523
2000 sex-ratio (male/female)	1.0651	0.0440	0.9896	1.1884
**Household**				
twochild (1 if having more than two children)	0.4684	0.4992	0	1
sheyes (1 if wife desiring more than two)	0.7941	0.4045	0	1
heyes (1 if husband desiring more than two)	0.8072	0.3946	0	1
number	1.4635	0.8301	0	7
income (in log function)	8.6801	4.4421	0	13.6412
**Wife**				
age	30.8135	4.0334	18	37
ideal number of children	1.9119	0.6285	0	7
education years	10.4615	3.9976	0	22
employment status (1 if currently employed)	0.8252	0.3799	0	1
hukou (1 if urban)	0.2439	0.4295	0	1
**Husband**				
age	31.3121	4.8175	16	61
ideal number of children	1.9416	0.6827	0	10
education years	10.3734	3.9628	0	22
employment status (1 if currently employed)	0.8442	0.3627	0	1
hukou (1 if urban)	0.2356	0.4245	0	1
**N**	2,054			

### Methods

To estimate the relationship between marital bargaining based on fertility preferences and second-child fertility decisions, we use the following OLS regression model:

$${\text{twochild}}_{\mathrm{jp}}=\beta_{0}+\beta_{1}\;{\text{sheyes}}_{\mathrm{jp}}+\beta_{2}\;{\text{heyes}}_{\mathrm{jp}}+\beta_{3}\;{\text{sheyes}}_{\mathrm{jp}}\times {\text{heyes}}_{\mathrm{jp}}+X_{\mathrm{jp}}\gamma +e_{\mathrm{jp}}$$ (1)

where *j* denotes household, *p* denotes province. *twochild _jp_* represents the fertility decision of family *j* in province *p* on whether to have more than two children, *sheyes _jp_* and *heyes _jp_* are the wife’s and husband’s preferences, respectively, for having more than two children. All three are binary variables. **X**_**jp**_ represents a vector of control variables, and *e _jp_* is the error term.

To estimate how one spouse’s fertility preferences might correlate to the other’s, we use the following two OLS regression models for husbands and wives:

$${\text{sheideal}}_{\mathrm{jp}}=\alpha_{0}+\alpha_{1}{\text{heideal}}_{\mathrm{jp}}+\alpha_{2}{\text{sexratio}}_{p}+\alpha_{3}{\text{heideal}}_{\mathrm{jp}}\times {\text{sexratio}}_{p}+X_{\mathrm{jp}}\theta +\epsilon_{\mathrm{jp}}$$ (2)

$${\text{heideal}}_{\mathrm{jp}}=\pi_{0}+\pi_{1}{\text{sheideal}}_{\mathrm{jp}}+\pi_{2}{\text{sexratio}}_{p}+\pi_{3}{\text{sheideal}}_{\mathrm{jp}}\times {\text{sexratio}}_{p}+X_{\mathrm{jp}}\sigma +\nu_{\mathrm{jp}}$$ (3)

where *sheideal _jp_* and *heideal _jp_* denote the ideal number of children for the wife and husband in household *j* in province *p*, and *sexratio _p_* represents sex ratio in province *p.* These variables are expressed as deviations from their respective sample means to make the data more comparable and interpretable. We do not use *sheyes _jp_* and *heyes _jp_*, because they represent only the preference for a second child and overlook the heterogeneity in fertility preferences, potentially failing to capture the actual preference. By definition, a person who prefers two children and another who prefers eight are assigned the same value when using *sheyes _jp_* or *heyes _jp_.* Therefore, we instead use *sheideal _jp_* and *heideal _jp_* to better capture an individual’s preference for family size and their association with their partner’s preference. *ε _jp_* and ν *_jp_* are error terms.

The results are presented sequentially. First, we examine the relationship of different fertility preferences with the family’s decision to have a second child. Then, we explore how one partner’s fertility preference might be associated with the other partner’s preference.

## Results

### Family fertility decision

The OLS results are reported in Table 2. Column (1) includes only explanatory variables and their interaction term; column (2) adds demographic characteristics; column (3) further controls for provincial fixed effects. All regression results show a statistically significant positive relationship between the second-child preferences of either spouse and the decision to have a second child in a family.

**Table 2.** Effects of marital bargaining over fertility preferences on second child fertility decision.

Dependent Variable	Whether Having Two Or More Children		
Variables	(1)	(2)	(3)
Sheyes	0.208 ***	0.175 ***	0.148 ***
	(0.0326)	(0.0325)	(0.0326)
Heyes	0.152 ***	0.133 ***	0.106 ***
	(0.0275)	(0.0278)	(0.0281)
Sheyes × Heyes	0.235 ***	0.199 ***	0.178 ***
	(0.0438)	(0.0426)	(0.0425)
Wife Employed		0.00182	-0.0035
		(0.0256)	(0.0254)
Husband Employed		-0.0735 ***	-0.0686 ***
		(0.0255)	(0.0255)
Wife Education Years		-0.0191 ***	-0.0197 ***
		(0.0032)	(0.0032)
Husband Education Years		-0.00938 ***	-0.0103 ***
		(0.0032)	(0.0032)
Wife Hukou		-0.0368	-0.0282
		(0.0263)	(0.0265)
Husband Hukou		-0.0483 *	-0.0505 *
		(0.0266)	(0.0270)
Wife Age		0.136 ***	0.128 ***
		(0.0268)	(0.0262)
Wife Age Square		-0.00168 ***	-0.00154 ***
		(0.0004)	(0.0004)
(Husband - Wife) Age		0.0113 ***	0.0112 ***
		(0.0030)	(0.0030)
Family Income		0.00263	0.00136
		(0.0022)	(0.0022)
Constant	0.0134 *	-2.131 ***	-2.153 ***
	(0.0077)	(0.3950)	(0.3990)
Province FE	NO	NO	YES
Observations	2,054	2,054	2,054
R-squared	0.203	0.339	0.376

Note: Robust standard errors in parentheses.

*** p<0.01.

** p<0.05.

* p<0.1.

We consider the situation with control for provincial fixed effects as the benchmark, as detailed in column (3). When a couple plans to have a second child, considering their fertility preferences, there are four possible outcomes: both agree to have the second one, both agree not to have it, the wife wants to have it but the husband disagrees, and the husband wants to have it but the wife disagrees. Our results show that when the couple reaches the agreement on the second child, it is associated with an increase in the likelihood of having a second child by more than 40% (0.148+0.106+0.178), the largest among the four outcomes. When a couple disagrees about having a second child, either spouse preferring a second child could be associated with an increased likelihood of having one. However, the wife’s preference tends to have a greater correlation with the household fertility decision. If only the wife agrees, her preference correlates to an increase in the household’s fertility probability by 14.8%, while if only the husband agrees, his preference correlates to an increase in the household’s fertility probability by 10.6%. All three estimators are significant at the 1% level and are robust across different specifications. This implies that to have a second child, both the wife and the husband need to cooperate to reach an agreement, especially when they have different second-child fertility preferences.

Moreover, the propensity for non-random marriage matching reinforces these findings, as individuals are more likely to pair with partners who share the same preference regarding having a second child. Table 3 illustrates this non-random distribution of marriage matching by fertility preference for the second child. For both men and women, the ratios of those preferring to have a second child to those who do not are approximately 4:1. If marriage matching were random, based on the sample size, we would expect to find 1,317 pairs of men and women both agreeing to have a second child, and 82 pairs agreeing not to have one. However, the actual numbers are higher: there are 1,459 and 224 households, respectively, that share the same fertility preference. Similarly, the households with couples share different fertility preferences are fewer than the expected under the assumption of random matching. This discrepancy supports the idea that couples match non-randomly based on fertility preferences.

**Table 3.** Non-Random distribution of marriage matching by fertility preference for the second child.

		Wife		
		Yes	No	Total
Husband	Yes	1,459 (1,317)	199 (341)	1,658
	No	172 (314)	224 (82)	396
Total		1,631	423	2,054

Note: Numbers in parentheses indicate matches in the random case.

Building on these observations, we are now interested in whether and how one spouse’s fertility preference associates with their partner’s.

### Individual fertility preference

In this section, we show correlation of wife’s and husband’s fertility preferences. The OLS results for wives and husbands are reported in Tables 4 and 5, respectively. In each table, columns (1) through (3) exclude self demographic characteristics, while columns (4) through (6) include them. Explanatory variables are added incrementally: we first introduce each spouse’s fertility preference in columns (1) and (4), then the provincial sex ratio in columns (2) and (5), and finally the interaction term between fertility preference and sex ratio in columns (3) and (6).

**Table 4.** Correlates of wife's fertility preference.

Dependent Variable	Wife's Ideal Number of Children					
Variables	(1)	(2)	(3)	(4)	(5)	(6)
Husband's ideal	0.410 ***	0.408 ***	0.414 ***	0.404 ***	0.402 ***	0.408 ***
	(0.0471)	(0.0471)	(0.0464)	(0.0459)	(0.0458)	(0.0453)
Sex ratio		0.374 *	0.302		0.380 **	0.311 *
		(0.1940)	(0.1860)		(0.1910)	(0.1840)
Husband's ideal × Sex ratio			0.546			0.522
			(0.5810)			(0.5650)
Husband Employed	-0.0515	-0.0527	-0.0518	-0.0505	-0.0517	-0.0509
	(0.0392)	(0.0392)	(0.0390)	(0.0388)	(0.0388)	(0.0386)
Husband Education Years	-0.0202 ***	-0.0207 ***	-0.0207 ***	-0.00730 *	-0.00776 *	-0.00787 *
	(0.0038)	(0.0038)	(0.0038)	(0.0044)	(0.0043)	(0.0043)
Husband Hukou	-0.0405	-0.0425	-0.0385	-0.00498	-0.00544	-0.00155
	(0.0333)	(0.0334)	(0.0331)	(0.0363)	(0.0362)	(0.0359)
Wife Employed				0.0441	0.0428	0.044
				(0.0325)	(0.0325)	(0.0325)
Wife Education Years				-0.0224 ***	-0.0223 ***	-0.0221 ***
				(0.0045)	(0.0045)	(0.0045)
Wife Hukou				-0.018	-0.0215	-0.022
				(0.0363)	(0.0363)	(0.0365)
Wife Age	-0.0108	-0.00787	-0.0077	-0.00576	-0.00276	-0.00277
	(0.0369)	(0.0368)	(0.0368)	(0.0369)	(0.0369)	(0.0369)
Wife Age Square	0.000276	0.000224	0.000217	0.000177	0.000125	0.000121
	(0.0006)	(0.0006)	(0.0006)	(0.0006)	(0.0006)	(0.0006)
(Husband - Wife) Age	0.00382	0.00366	0.00357	0.0025	0.00229	0.00225
	(0.0041)	(0.0041)	(0.0041)	(0.0041)	(0.0041)	(0.0041)
Family Income	-0.00525 *	-0.00536 *	-0.00536 *	-0.00329	-0.00339	-0.00341
	(0.0029)	(0.0029)	(0.0029)	(0.0029)	(0.0029)	(0.0029)
Constant	0.373	0.347	0.345	0.356	0.329	0.328
	(0.5580)	(0.5570)	(0.5570)	(0.5560)	(0.5550)	(0.5550)
Observations	2,054	2,054	2,054	2,054	2,054	2,054
R-squared	0.264	0.265	0.267	0.275	0.277	0.278

Note: Robust standard errors in parentheses.

The sample is limited to females aged below 37.

*** p<0.01.

** p<0.05.

* p<0.1.

**Table 5.** Correlates of husband's fertility preference.

Dependent Variable	Husband's Ideal Number of Children					
Variables	(1)	(2)	(3)	(4)	(5)	(6)
Wife's ideal	0.497 ***	0.495 ***	0.502 ***	0.491 ***	0.489 ***	0.495 ***
	(0.0315)	(0.0316)	(0.0319)	(0.0311)	(0.0311)	(0.0315)
Sex ratio		0.26	0.193		0.302	0.237
		(0.2280)	(0.2270)		(0.2250)	(0.2240)
Wife's ideal × Sex ratio			0.702 *			0.668 *
			(0.3890)			(0.3870)
Wife Employed	-0.0426	-0.0433	-0.0418	-0.0425	-0.0434	-0.042
	(0.0388)	(0.0388)	(0.0388)	(0.0388)	(0.0387)	(0.0387)
Wife Education Years	-0.00527	-0.00545	-0.00505	0.00356	0.00356	0.00379
	(0.0040)	(0.0040)	(0.0040)	(0.0050)	(0.0050)	(0.0051)
Wife Hukou	-0.110 ***	-0.113 ***	-0.112 ***	-0.0830 *	-0.0857 **	-0.0852 **
	(0.0391)	(0.0389)	(0.0389)	(0.0435)	(0.0432)	(0.0432)
Husband Employed				0.0161	0.015	0.0146
				(0.0410)	(0.0409)	(0.0409)
Husband Education Years				-0.0151 ***	-0.0154 ***	-0.0152 ***
				(0.0051)	(0.0051)	(0.0051)
Husband Hukou				-0.0251	-0.0255	-0.0257
				(0.0458)	(0.0458)	(0.0459)
Wife Age	0.0446	0.0464	0.0453	0.0548	0.0571	0.0559
	(0.0391)	(0.0392)	(0.0393)	(0.0400)	(0.0401)	(0.0401)
Wife Age Square	-0.000687	-0.000718	-0.0007	-0.000872	-0.000912	-0.000892
	(0.0006)	(0.0006)	(0.0006)	(0.0007)	(0.0007)	(0.0007)
(Husband - Wife) Age	0.00732 *	0.00716 *	0.00753 *	0.00756 *	0.00739 *	0.00775 *
	(0.0039)	(0.0039)	(0.0039)	(0.0041)	(0.0041)	(0.0042)
Family Income	-0.00644 *	-0.00654 *	-0.00651 *	-0.00523	-0.00531	-0.00529
	(0.0035)	(0.0035)	(0.0035)	(0.0035)	(0.0035)	(0.0035)
Constant	-0.542	-0.558	-0.55	-0.639	-0.659	-0.649
	(0.5740)	(0.5740)	(0.5760)	(0.5810)	(0.5820)	(0.5830)
Observations	2,054	2,054	2,054	2,054	2,054	2,054
R-squared	0.25	0.251	0.252	0.255	0.255	0.257

Note: Robust standard errors in parentheses.

The sample is limited to females aged below 37.

*** p<0.01.

** p<0.05.

* p<0.1.

We consider the situation including self demographic characteristics as the benchmark, as detailed in column (6) of both tables. The results for both husbands and wives show that if they prefer more children, their partners also prefer more children. All estimators are significant at the 1% level. Specifically, if a husband’s ideal number of children is one more than the average male preference, his wife will likely prefer 0.4 more children than the average female preference. In contrast, if a wife’s ideal number of children exceeds the average female preference by one, her husband will likely prefer 0.5 more children than the average male preference.

Males and females exhibit asymmetry when considering the provincial-level sex ratio. We use the sex ratio for those aged between 20 and 39 in a province to capture the potential population in the marriage market. A larger sex ratio, indicating more males than females, serves as a proxy for women’s higher bargaining power. This is because a higher sex ratio intensifies competition among males for potential female partners, thereby enhancing women’s bargaining position in the marriage market and in family decisions. In provinces where the sex ratio is one percentage point higher than the national average, a wife’s preference affects her husband’s preference much more strongly. Conversely, in such provinces, a husband’s preference affects his wife’s preference in a similar manner when the sex ratio becomes higher. More specifically, if the wife prefers one more child than the average female ideal number, then her husband will prefer 0.5 (0.495+0.668×1%) more children than the average male ideal number. Conversely, the husband’s fertility preference is not associated with his wife’s preference, regardless of the sex ratio in their resident province. This suggests that women’s increased bargaining power influences assortative matching, due to their smaller number in the marriage market. The greater bargaining power enables women to match with partners whose fertility preferences are associated with theirs.

## Conclusions

In this paper, we examine fertility decisions regarding the second child, focusing on differing marital fertility preferences by utilizing data from the 2018 China Family Panel Studies survey and applying Ordinary Least Squares regression models. We further explore assortative matching based on fertility preferences, using the provincial-level sex ratio from 2020 for the population aged between 20 and 39 as a proxy for women’s bargaining power, which captures the potential population in the marriage market. Our findings reveal that having a second child requires cooperation between the wife and the husband, especially when they have differing fertility preferences. Additionally, our research shows that marriage matching is not random; individuals are more likely to pair with partners with the same preference for having a second child. Further analysis demonstrates that females with greater bargaining power correlate positively with their husbands’ ideal family size, whereas males do not have a similar correlation. This paper contributes to the literature on fertility decisions from the perspective of the bargaining process within the Chinese context, as well as preference matching and sorting.

## Data Availability

We are not allowed to distribute the original data since the data was collected by Peking University and the agreement with Peking University does not allows us to distribute the data. However, we provide the link to obtain the data from Pekin University and we provide the code to replicate our results. Our data set(code) is a is available at Open Science Framework (OSF) ( Zhuang and Naito (2024)): https://doi.org/10.17605/OSF.IO/F5DPZ. The original dataset used in this study is available from the following URL: https://opendata.pku.edu.cn/dataverse/CFPS?language=en Researchers who want to replicate our results can obtain the data set by proposing a research proposal to Peking University through the above website. The code to replicate our results are available on the above OSF domain. The findings, interpretations, and conclusions expressed in this article are entirely those of the authors and should not be attributed in any manner to Peking University, to its affiliated organizations. Zhuang and Naito (2024) on OSF contains the following data: data.do: Stata file for data cleaning tables.do: Stata file for figures and regression analysis Tables.xlsx: Excel file for all tables The above data are available under the terms of the Creative Commons Zero “No rights reserved” data waiver (CC0 1.0 Public domain dedication). Repository: STROBE checklist for ‘Marital Bargaining and Assortative Matching on Fertility Preference: Evidence based on Cross-sectional Data in China’ is available at https://doi.org/10.17605/OSF.IO/2Z8HY. Ethics and consent: we obtained the approval to use this data on August 9, 2021 from Peking University. Since this data is in the public domain and it is widely used, the review thorough the ethical committee at the University of Tsukuba was not needed. The original data collection project by Peking University was approved by the Biomedical Ethics Committee of Peking University, Beijing, China. The ethical approval number is IRB00001052-14010. We obtained the permission to publish this research on June 2 ^nd^ 2024 from Peking University.
