# Marital Bargaining and Assortative Mating on Fertility Preference : Evidence based on Cross-sectional Data in China

**DOI:** 10.12688/f1000research.151196.4

**Published:** 2025-07-30

**Authors:** Meiyi Zhuang, Hisahiro Naito

**Affiliations:** 1 Graduate School of Humanities and Social Sciences, University of Tsukuba, Tsukuba Tennodai 1-1-1, Ibaraki, 305-8571, Japan; 2 Graduate School of Humanities and Social Sciences, University of Tsukuba, Tsukuba Tennodai 1-1-1, Ibaraki, 305-8571, Japan

**Keywords:** Fertility preference, assortative matching, marriage, bargaining, male-female ratio, China

## Abstract

**Background:**

Behaviors regarding child-bearing are among the most consequential ones within families, as child-rearing necessitates the active involvement of both partners. This dynamic suggests that individuals may seek partners with similar fertility preferences, leading to assortative mating based on these shared preferences.

**Data and Methods:**

This study investigates second-child fertility outcomes using data from the 2018 China Family Panel Studies and applies the Ordinary Least Squares (OLS) regression. Furthermore, it examines assortative matching based on fertility preferences, utilizing the 2020 provincial-level sex ratio for individuals aged 20–39 as a proxy for women’s bargaining power in the marriage market.

**Results:**

The results, based on a sample of 2,351 household-level observations, indicate that spouses’ preferences are complementary. When a husband (or wife) desires a second child, their own preference has a stronger impact on the actual outcome to have of having a second child if their partner also expresses the same desire. Additionally, the findings reveal evidence of assortative mating in terms of preferences for the desired number of children. Moreover, in provinces with higher male-to-female sex ratios—indicating stronger bargaining power for women—a wife’s preference is more strongly aligned with her husband’s preference. For men, however, a husband’s preference aligns with his wife’s preference regardless of the sex ratio, suggesting that men’s bargaining power in the marriage market is relatively weak.

**Conclusions:**

The findings have two key implications. First, shared fertility preferences between spouses show a complementary effect, increasing the likelihood of having a second child. Second, marriage matching is not random, as individuals are more likely to partner with those who share similar fertility preferences. Additionally, women with greater bargaining power are positively associated with their husbands’ desired family size.

## Introduction

Preferences shape both collective decisions and individual behaviors. The decisions of a group are often influenced by the preferences of its members, becoming more complex when these preferences diverge. Consequently, individuals tend to gravitate toward groups with shared preferences to minimize conflicts and avoid the need for extensive negotiation.

Within families, behaviors regarding childbearing—whether to have children and how many—are among the most consequential, as raising children requires the cooperation and support of both partners. This dynamic suggests that individuals may seek partners with similar fertility preferences, leading to assortative mating based on these shared preferences.

Recent studies emphasize the importance of both spouses’ fertility preferences and mutual agreement on the desired number of children in determining actual fertility outcomes ( Rasul, 2008; Doepke and Kindermann, 2019). The significance of shared spousal preferences suggests that men and women in the marriage market may have strong incentives to select partners with similar fertility preferences. When spouses share similar views on family size, they can potentially avoid the need for extensive bargaining. Consequently, in contexts where preferences regarding the number of children vary, assortative matching based on fertility preferences naturally emerges in the marriage market. Therefore, it is essential to examine how bargaining dynamics and preference-based matching shape family size outcomes.

Understanding assortative mating based on fertility preferences can have significant policy and institutional implications. For example, the one-child policy began to be relaxed in 2011, and, interestingly, starting in 2013, marriage rates began to decline. ^1^ Before the relaxation of the one-child policy, couples had only two options: to have no children or to have one child. This limited choice reduced the likelihood of disagreement over the desired number of children when men and women sought partners. However, after the policy was relaxed, couples had more options regarding family size. This broader range of preferences may have increased the chances of disagreement between potential partners on the desired number of children, making it more difficult to find an ideal match. This dynamic could have contributed to the observed decline in marriage rates.

In this study, we first examine fertility outcomes regarding the second child, focusing on both spouses’ fertility preferences, using data from the 2018 China Family Panel Studies survey and applying the Ordinary Least Squares (OLS) regression model. Second, we explore assortative matching based on fertility preferences, using the provincial-level sex ratio as a proxy for women’s bargaining power. To this end, we collect data on the number of males and females aged 20-39 from the 2020 China Population Census Yearbook and calculate the sex ratio by dividing the number of males by the total population of both sexes. This approach captures the potential population in the marriage market.

We find that spouses’ preferences are complementary. When a husband (or wife) desires a second child, their own preference is more strongly associated with the realization of a second birth if their partner also expresses a desire for a second child. This effect is particularly evident among couples with a highly educated wife or those with a male-hukou advantage (i.e., the husband’s hukou status is at least equal to the wife’s). Additionally, we find that marriage matching is not random, as individuals are more likely to partner with those who share similar fertility preferences.

Furthermore, when wives have greater bargaining power, their ideal number of children tends to align more closely with their husbands’ preferences, whereas no such pattern is observed for men. This suggests that in provinces with a smaller ratio of women, women can exert strong bargaining power and influence the choice of their partners. For men, however, in provinces with a smaller ratio of men, they cannot similarly enhance their bargaining power or influence their partner’s choices. This asymmetry highlights the fact that a smaller ratio of men does not enhance men’s bargaining power in the Chinese marriage market, as the overall ratio of men remains significantly larger in China.

**Figure 1.**
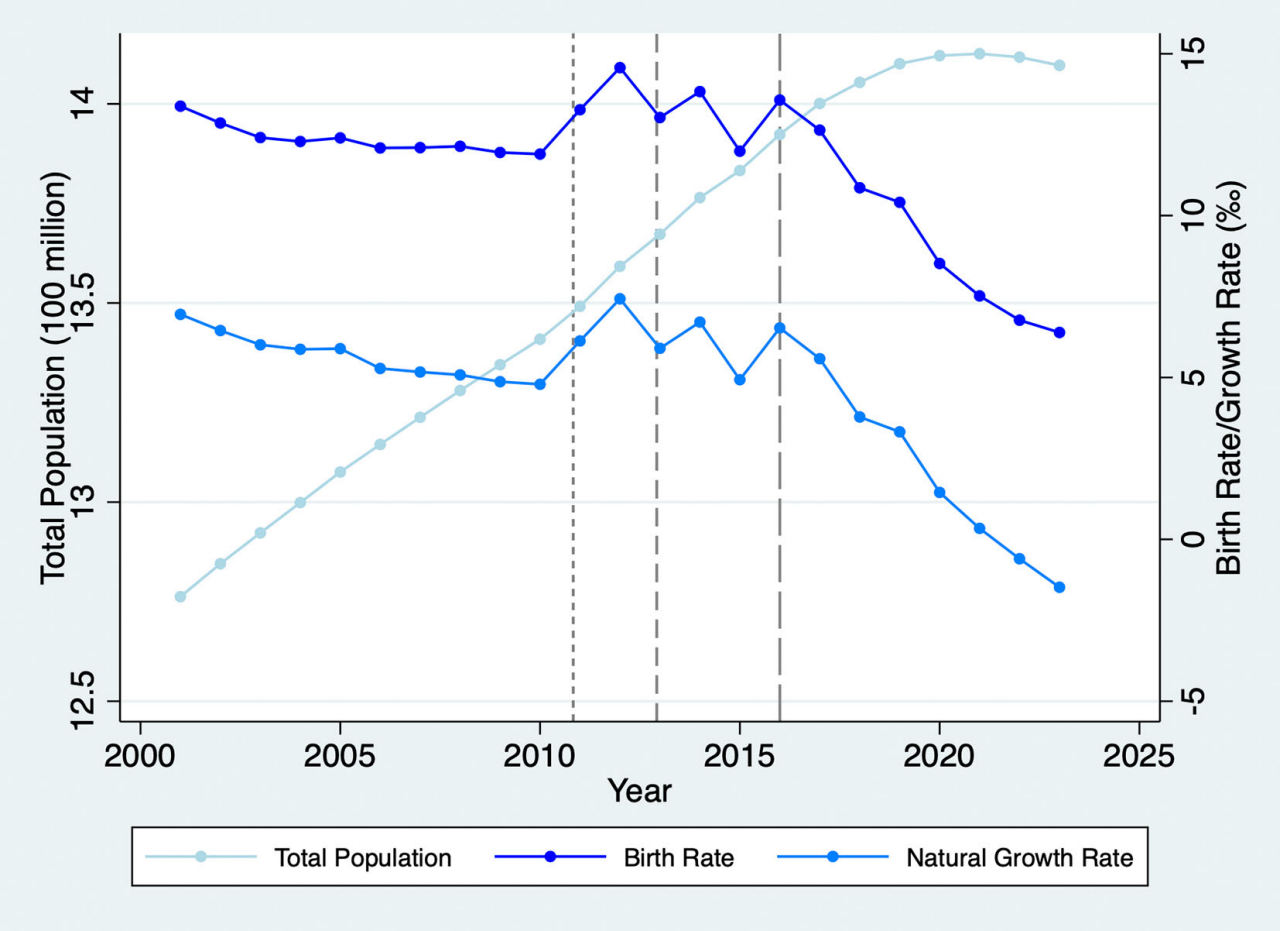
Total Population, Birth, and Natural Growth Rates in China, 2001-2023. Note: The short-dash, dash, and long-dash lines denote the starting times of the Double-Single, Selective, and Universal Two-Child Policies, respectively.

We study fertility preferences and assortative mating using the Chinese context for two reasons. First, while the Universal Two-Child Policy encourages families to consider having a second child, China’s birth rate has continued to decline to historic lows (as illustrated in Figure 1), with most couples continuing to have only one child.

Second, women’s bargaining power within families has increased because of China’s rapid economic development and the long-standing male-skewed sex ratio, which empowers them with greater influence in the marriage market. Consequently, women who hold strong preferences for the ideal number of children are more likely to seek partners who share or align with their fertility preferences. These conditions render China an ideal case to explore whether assortative mating based on fertility preferences is occurring in the marriage market, which links marriage rates and birth rates.

This study contributes to the literature on assortative mating. Previous studies have primarily examined assortative mating from the perspectives of personal traits (e.g., Dupuy and Galichon, 2014; Chiappori et al., 2024) or socioeconomic characteristics (e.g., social class in Abramitzky et al., 2011; education in Eika et al., 2019; skill in Persson, 2020). This study introduces a novel perspective by exploring assortative mating based on fertility preferences, thereby linking marriage and fertility outcomes. We find that in the Chinese marriage market, males and females select partners based on their preferences for the ideal number of children, even after controlling for demographic characteristics such as urban residence, education, age, and province fixed effects. We also examine how the bargaining power of men and women is associated with this assortative mating.

Our findings show that the ratio of women to men—a proxy for the bargaining power of females and males—is differently associated with partner selection for men and women. In provinces with a lower ratio of women, women’s preferences show a stronger association with their partner’s preferences, while men do not exhibit a similarly strong association with those of their partners.

To the best of our knowledge, no existing study has investigated the impact of fertility preferences on assortative mating. This study aims to provide evidence on this topic, using the Chinese case as an example.

Nonetheless, our study has a few limitations. Owing to constraints in the survey design, the sample size is limited to approximately 2,000 observations. This may impact the generalizability of our results, although it is, to the best of our knowledge, the most appropriate dataset available for studying the Chinese case. In addition, due to our cross-sectional dataset, our analysis reflects associations rather than causal relationships. In future work, we aim to explore potential exogenous variations to better identify causal effects.

The remainder of the article is organized as follows: Section 2 reviews related literature. Section 3 presents the data and empirical strategy used for the empirical analysis. Section 4 explores how fertility outcomes are related to both spouses’ fertility preferences, how one spouse’s preference is associated with their partner’s, and heterogeneity analysis by educational levels and hukou types. Section 5 discusses the findings and policy implications. Section 6 concludes the paper.

## Literature review

### Fertility decisions

Family decisions involve several resource allocation problems, from how to divide income between consumption and saving, to how to allocate time between work and leisure, and from whether and whom to marry, to whether and when to have children.

There are three strands of theoretical household decision-making models. The first model is the unitary model, which assumes a single and unified household utility, where, typically, the male (husband or father) makes all major decisions ( Becker, 1960, 1973). The second model is the cooperative model, which allows for heterogeneous utility functions and preferences among spouses. This model has two frameworks: the explicit bargaining model, which incorporates the negotiation process based on each spouse’s outside options, as proposed by Manser and Brown (1980) and McElroy and Horney (1981); and the collective model, which internalizes the negotiation process, as introduced by Chiappori (1988, 1992). The third model is the non-cooperative model, which relaxes the assumption of Pareto efficient equilibria, and the threat point is non-cooperation within the marriage rather than divorce ( Lundberg and Pollak, 1993, 1994). Commitment plays a crucial role in the latter two strands ^2^. The empirical research on fertility decisions follows three strands corresponding to the three types of theoretical models.

Within the unitary model framework, the quantity–quality theory often uses twins as a natural experiment, showing that an increase in family size decreases the children’s educational attainment ( Rosenzweig and Wolpin, 1980; Rosenzweig and Zhang, 2009). Additionally, extensive literature explores fertility from women’s perspectives, showing that the relationship between income, education, and fertility has shifted from negative to positive ( Butz and Ward, 1979; Ahn and Mira, 2002; Baudin et al., 2015) ^3^.

Although it is difficult to incorporate cooperation into an empirical model, examining marriage outcomes, such as divorce or specialization within marriage, and partners’ interactions can help infer how fertility is determined. Rasul (2008) finds that Malay couples’ fertility preferences equally and positively influence outcomes, indicating cooperative bargaining, supported by high divorce rates for Malay couples, which serve as threat points in bargaining models. Bankole (1995) shows that Nigerian couples generally cooperate on fertility decisions, with both spouses’ preferences equally influencing outcomes. However, the husband’s preferences dominate early (fewer than four children), while the wife’s dominate later, suggesting that cooperation shifts over the marriage span and is not always consistent.

Regarding non-cooperative empirical studies, Ashraf et al. (2014) and Ashraf et al. (2020) use Zambian field experiments to show that asymmetric information between spouses leads to non-cooperative fertility decisions. Concealing contraceptive use reduces fertility but lowers women’s well-being (2014), while asymmetric maternal health knowledge affects communication and spousal transfers, highlighting inefficiencies in fertility choices (2020). Field (2003) links Peru’s property rights reform to reduced fertility, as women’s economic independence reduced the reliance on children for old-age support, aligning with the non-cooperative model by enhancing women’s control over fertility decisions ^4^.

### Assortative mating

Lafortune (2013) studies how the male-biased sex ratio, driven by the influx of same-ethnic immigrants, affects the marriage patterns of second-generation Americans, who tend toward endogamy. Addressing concerns that influx locations might be related to labor market opportunities, which could also affect second-generation Americans, the study uses historical immigrant networks as an instrument. It finds that higher sex ratios lead to increased male education and exogamy, while females remain more endogamous. Persson (2020) studies a 1989 Swedish insurance reform that reduced marital benefits for women, leading to an increase in skill-based assortative mating. Before the reform, the insurance system subsidized skill-mismatched marriages, providing financial incentives for lower-skilled women to pair with higher-skilled husbands. After the reform, couples prioritized maximizing marital surplus, resulting in more skill-similar pairings. Abramitzky et al. (2011) use World War I as a natural experiment in France, showing that a decline in the male population increased male bargaining power, enabling them to “marry up” into higher social classes categorized by occupation. Considering the potential reverse causality that marriage outcomes could also affect the sex ratio, they instrument the sex ratio with the regional military mortality rate, which provides geographic variation in the sex ratio. Fisman et al. (2006) use a dating field experiment to show the asymmetric preferences between males and females, while Banerjee et al. (2013) show the persistence of caste in the Indian marriage market using matrimonial advertisement data, attributing it to a frictionless marriage market. Recent work by Goñi (2022) uses a natural experiment that introduced search costs to overcome the limitations of survey data. The interruption of the London Season (an occasion where aristocrats met) during 1861–1863 made search and mating with partners with similar socioeconomic traits more difficult. The study finds that increased search costs discourage assortative mating by social class.

### Applications to the Chinese case

In China—one of the most populous countries facing a decline in fertility—quantitative research on marital bargaining remains scarce ^5^. Peng (2020) qualitatively highlights the role of negotiations in fertility through interviews with 53 urban couples. However, the empowerment of Chinese women in recent decades has likely influenced household decisions in this representative developing country.

Regarding assortative mating, Chinese studies on educational assortative mating show that its effect is significant and steadily increasing ( Dong and Xie, 2023), while it is less likely to occur in remarriages ( Hu and Qian, 2019). Sun and Zhang (2020) find a positive effect of housing prices on the father-in-law’s years of schooling.

Chinese studies also emphasize the role of the hukou status, which categorizes residents into urban and rural groups. When local hukou is restricted, assortative mating based on hukou increases ( Qian and Qian, 2017). Conversely, when it is unrestricted, intermarriage becomes more common, reducing assortative mating ( Han et al., 2015; Hu, 2024), which, in turn, exacerbates inequality ( Wang and Schwartz, 2018). However, no study, either in China or globally, has examined assortative mating based on fertility preferences.

This study addresses these research gaps by providing novel insights into how fertility outcomes are associated with the fertility preferences of both spouses, and how preferences regarding a second child are aligned between men and women, using the Chinese case as an example.

## Methods

### Data set

The primary dataset utilized in this study is the China Family Panel Studies (CFPS)—a nationally representative longitudinal sample of Chinese communities, families, and individuals ( Xie and Hu, 2014). The survey includes information on economic activities, educational outcomes, family relationships, migration, and health. Initiated by the Institute of Social Science Survey (ISSS) at Peking University, the baseline survey was conducted in 2010, with follow-up full-sample surveys conducted biennially in 2012, 2014, 2016, 2018, and 2020. Our analysis uses the cross-sectional data from the 2018 CFPS, which includes approximately 44,000 individuals in 15,000 households from more than 900 counties and cities within 31 provinces, municipalities, and autonomous regions.

The CFPS employs a multi-stage probability sampling strategy to ensure a representative sample of the Chinese population. In the first and second stages, it stratifies and selects provinces and counties using official administrative divisions. Recognizing the geographic disparities inherent in China’s economic development, the design emphasizes geographic representation. In the third stage, households within the chosen counties are selected based on a systematic sampling method, targeting 25 households per county to fulfill the study’s requirements.

**Figure 2.**
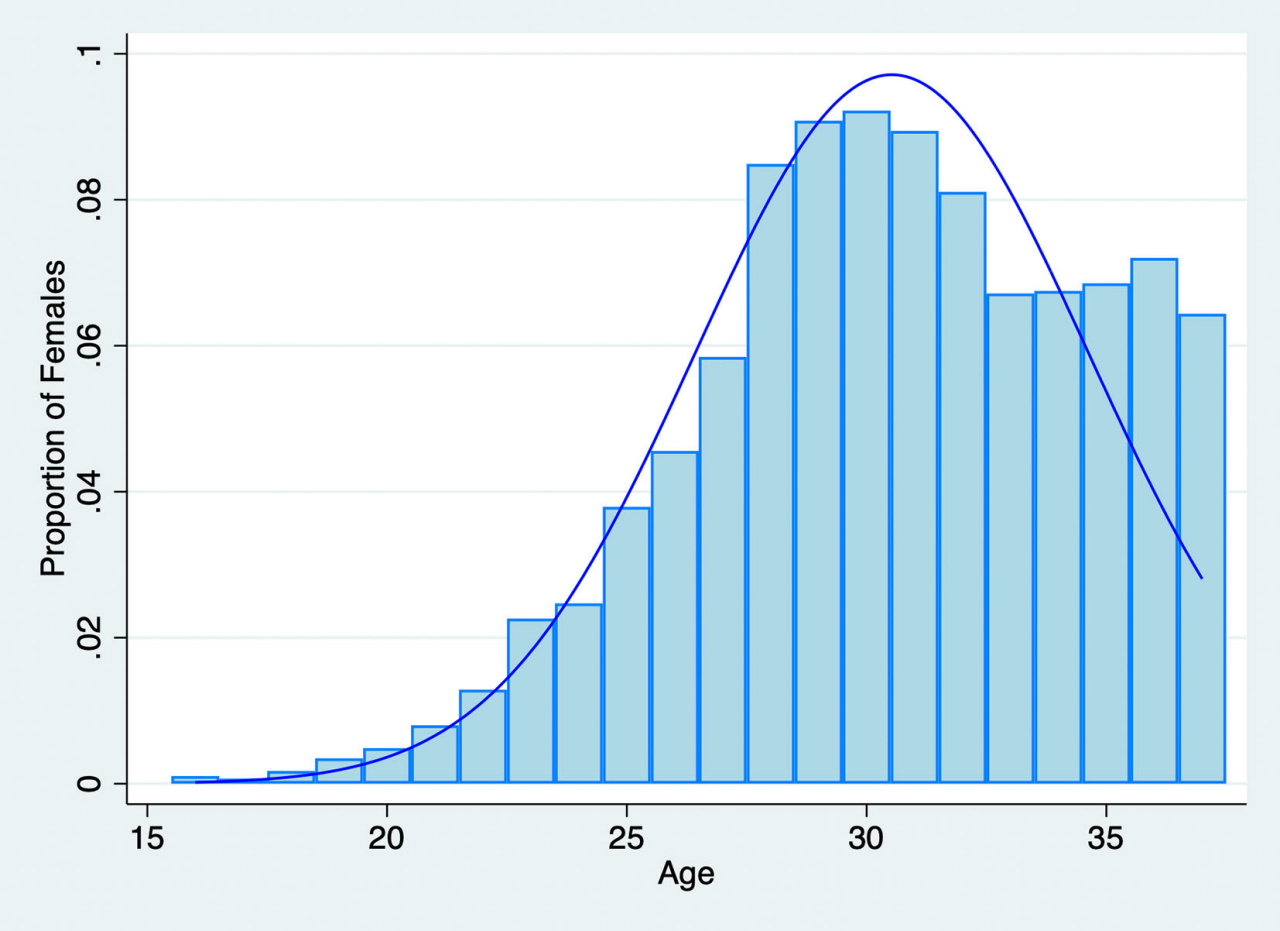
Distribution of Female Age in the Sample, *N*=2,351.

Since we seek to understand how fertility outcomes are associated with both spouses’ fertility preferences, we initially restricted the sample to individuals who responded to questions regarding their ideal number of children. In the 2018 CFPS dataset, 16,268 out of 18,802 females and 16,214 out of 18,552 males provided their responses. Considering both the husband’s and the wife’s preferences, we further constructed a household-level sample that includes information on both spouses. We limited the sample to couples where both spouses responded to the question about the “ideal number of children,” as this measures their fertility preference for a second child. We also restricted the age range for women to be equal to or less than 37, corresponding to the possible reproductive age. The distribution of female ages in the sample is illustrated in Figure 2. Ultimately, the sample comprises 2,351 household observations with detailed information on both husbands and wives. We obtained the permission to use this data set on August 9, 2021.

The main outcome variable in this study is whether the household has two or more children. This variable is derived from the number of children in the family. Since the CFPS does not directly provide complete information on family size, we generated this variable using information available for each child. If both spouses reported their ideal number of children but the actual number of children is missing, we replaced the missing values with 0. In the sample, 49.43% of households have more than two children. The key explanatory variables are the fertility preferences of both spouses for the second child, derived from their reported ideal number of children. In the sample, 79.41% of wives and 80.82% of husbands prefer more than two children. In the second part, the outcome variables are individual fertility preferences, measured by their ideal number of children. On average, both females and males desire fewer than two children. Control variables include both spouses’ employment status (employed or unemployed, without distinguishing between being out of the labor force or unemployed), years of education, age, hukou type (rural or urban), and family income. We assigned the income to be zero if the individuals are currently unemployed. Family income in logarithmic form is calculated by summing the incomes of both spouses and adding one, to avoid issues with missing values when both spouses have no income.

The previous literature has shown, both empirically and theoretically, that a skewed sex ratio increases women’s bargaining power in the marriage market ( Angrist, 2002; Chiappori et al., 2002; Bulte et al., 2015). As this study focuses on the marital bargaining process regarding family fertility outcomes, we use the provincial-level sex ratio as a proxy for bargaining power. We collected data on the number of males and females aged 20-39 from the China Population Census Yearbooks for 2000, 2010, and 2020. The age range of 20-39 captures the potential population in the marriage market. There is no information available for 2018, as the census is conducted every ten years. We calculated the sex ratio as a percentage by dividing the number of males by the total number of males and females. Given that the statistics are similar across these three census waves, we report only the results using the 2020 sex ratios in this paper. In 2020, the average sex ratio for the population aged 20-39 across 31 provinces was 51.89%, implying there are more males than females in the marriage market. The detailed summary statistics for all variables are presented in Table 1.

**Table 1.** Summary statistics.

Variables	Mean	SD	Min	Max
**Province**				
2020 sex ratio (male / (female + male)) × 100%	51.8876	1.2039	49.6353	54.7222
2010 sex ratio (male / (female + male)) × 100%	51.0093	1.3550	48.8112	55.6014
2000 sex ratio (male / (female + male)) × 100%	51.5542	1.0151	49.7391	54.3055
**N**	31			
**Household**				
twochild (1 if having more than two children)	0.4943	0.5001	0	1
sheyes (1 if wife desiring more than two)	0.7941	0.4044	0	1
heyes (1 if husband desiring more than two)	0.8082	0.3938	0	1
number of children	1.5155	0.8083	0	7
income (in log function)	8.6901	4.4393	0	13.6412
**Wife**				
age	30.5249	4.1262	16	37
ideal number of children	1.8937	0.5847	0	5
education years	10.1821	4.1448	0	18
employment status (1 if currently employed)	0.7244	0.4469	0	1
hukou (1 if urban)	0.2293	0.4204	0	1
income (in log function)	5.2913	5.1367	0	13.1224
**Husband**				
age	32.6453	5.0869	16	62
ideal number of children	1.9536	0.7050	0	10
education years	10.4032	3.8413	0	22
employment status (1 if currently employed)	0.9604	0.1950	0	1
hukou (1 if urban)	0.2488	0.4324	0	1
income (in log function)	7.5942	4.8648	0	13.6412
**N**	2,351			

## Methods

To estimate the relationship between both spouses’ fertility preferences and second-child fertility outcomes, we use the following OLS regression model:

$${\text{twochild}}_{\mathrm{jp}}=\beta_{0}+\beta_{1}{\text{sheyes}}_{\mathrm{jp}}+\beta_{2}{\text{heyes}}_{\mathrm{jp}}+\beta_{3}{\text{sheyes}}_{\mathrm{jp}}\times {\text{heyes}}_{\mathrm{jp}}+X_{\mathrm{jp}}\Gamma +\beta_{p}+e_{\mathrm{jp}}$$ (1)

where *j* denotes household, *p* denotes province. twochild _*jp*_ represents the fertility outcome of family *j* in province *p* on whether to have more than two children, sheyes *_jp_* and heyes *_jp_* are the wife’s and husband’s preferences, respectively, for having more than two children. All three are binary variables. *X*_*jp*_ represents a vector of control variables, $\beta_{p}$ represents the province fixed effect, and *e _jp_* is the error term. The coefficient of our interest is $\beta_{3}$ . It indicates how the marginal effect of an individual’s own preference for having a second child is associated with their partner’s preference when the partner also desires a second child. If $\beta_{3}$ is positive, it suggests that when both the wife and husband share the same preference, it becomes easier to have a second child owing to reduced negotiation and increased cooperation.

To estimate how one spouse’s fertility preferences might be correlated with the other’s, we use the following two OLS regression models for husbands and wives:

$${\text{sheideal}}_{\mathrm{jp}}=\alpha_{0}+\alpha_{1}{\text{heideal}}_{\mathrm{jp}}+\alpha_{2}{\text{heideal}}_{\mathrm{jp}}\times {\text{sexratio}}_{p}+X_{\mathrm{jp}}\Theta +\alpha_{p}+\epsilon_{\mathrm{jp}}$$ (2)

$${\text{heideal}}_{\mathrm{jp}}=\pi_{0}+\pi_{1}{\text{sheideal}}_{\mathrm{jp}}+\pi_{2}{\text{sheideal}}_{\mathrm{jp}}\times {\text{sexratio}}_{p}+X_{\mathrm{jp}}\Phi +\pi_{p}+\eta_{\mathrm{jp}}$$ (3)

where sheideal *_jp_* and heideal *_jp_* denote the ideal number of children for the wife and husband in household *j* in province *p*, and sexratio *_p_* represents sex ratio in province *p.* These variables are expressed as deviations from their respective sample mean to render the data more comparable and interpretable. We do not use sheyes *_jp_* and heyes *_jp_*, because they represent only the preference for a second child and overlook the heterogeneity in fertility preferences, potentially failing to capture the actual preference. By definition, a person who prefers two children and another who prefers eight are assigned the same value when using sheyes *_jp_* or heyes *_jp_.* Therefore, we use sheideal *_jp_* and heideal *_jp_* to better capture an individual’s preference for family size and their association with their partner’s preference. $\alpha_{p}$ and $\pi_{p}$ are province fixed effects, ε *_jp_* and $\eta_{\mathrm{jp}}$ are error terms.

The results are presented sequentially. First, we examine the relationship of different fertility preferences with the family’s outcome to have a second child. Thereafter, we explore how one partner’s fertility preference might be associated with the other partner’s preference.

## Results

### Family fertility outcome

The OLS results are reported in Table 2. Column (1) includes only explanatory variables and their interaction term; column (2) adds demographic characteristics; column (3) further controls for provincial fixed effects. All regression results show a statistically significant positive relationship between the second-child preferences of either spouse and the fertility outcome regarding a second birth.

**Table 2.** Effects of marital bargaining over fertility preferences on second child fertility outcome.

Dependent variable	Whether having two or more children		
Variables	(1)	(2)	(3)
Sheyes	0.243 ***	0.175 ***	0.150 ***
	(0.0322)	(0.0319)	(0.0318)
Heyes	0.128 ***	0.112 ***	0.0929 ***
	(0.0244)	(0.0253)	(0.0260)
Sheyes × Heyes	0.257 ***	0.240 ***	0.211 ***
	(0.0413)	(0.0403)	(0.0402)
Controls	No	Yes	Yes
Province FE	No	No	Yes
Observations	2,351	2,351	2,351
R-squared	0.229	0.370	0.403

Note: The estimated coefficients of control variables are displayed in Table A1. Robust standard errors in parentheses.

*** p<0.01,

** p<0.05,

* p<0.1.

We consider the situation with provincial fixed effects as the benchmark, as detailed in column (3). Our results show that when the couple has the same preference for having the second child, it is associated with an increase in the likelihood of having a second child by 45.39% (0.150+0.0929+0.211). When only the wife prefers having two or more children, the likelihood of the couple having a second child increases by 15 percentage points, holding other factors constant; similarly, when only the husband prefers having a second child, the likelihood increases by approximately 9.3 percentage points. The interaction term indicates the complementary effect between the wife’s and husband’s preferences. When a husband (wife) desires a second child, the association between their own preference and the actual fertility outcome becomes more pronounced if their partner also expresses a desire for a second child. For example, when the husband prefers a second child, the wife’s marginal effect on the likelihood of having two or more children increases from 0.15 to 0.36 (0.15+0.21). Similarly, when the wife strongly prefers a second child, the husband’s marginal effect increases from 0.093 to 0.30 (0.093+0.21). All estimates are significant at the 1% level and are robust across different specifications. These findings imply that when both spouses prefer to have a second child, reduced negotiation and bargaining enhance the marginal effect of one spouse’s preference on the actual outcome of having a second child, thus highlighting the importance of assortative mating.

Moreover, the propensity for non-random marriage matching reinforces these findings, as individuals are more likely to pair with partners who share the same preference regarding having a second child. Table 3 illustrates this non-random distribution of marriage matching by fertility preference for the second child. For both men and women, the ratios of those preferring to have a second child to those who do not are approximately 4:1. If marriage matching were random, based on the sample size, we would expect to find 1,509 pairs of men and women both agreeing to have a second child, and 93 pairs agreeing not to have one. However, the actual numbers are higher: there are 1,670 and 254 households, respectively, that share the same fertility preference. Similarly, the households where couples share different fertility preferences are fewer than expected under the assumption of random matching. This discrepancy supports the idea that couples match non-randomly based on fertility preferences.

**Table 3.** Non-Random distribution of marriage matching by fertility preference for the second child.

		Wife		
		Yes	No	Total
**Husband**	Yes	1,670 (1,509)	230 (391)	1,900
	No	197 (358)	254 (93)	451
**Total**		1,867	484	2,351

Note: Numbers in parentheses indicate matches in the random case.

Building on these observations, we are now interested in whether, and how, one spouse’s fertility preference associates with their partner’s.

### Individual fertility preference

In this section, we examine how—and to what extent—one partner’s fertility preference is associated with the other’s. The OLS results for husbands and wives are reported in Tables 4 and 5, respectively. In each table, the explanatory variables include one partner’s fertility preferences and the interaction term between fertility preference and the sex ratio, which serves as a proxy for female bargaining power. Since we control for province fixed effects, provincial-level sex ratio estimates are automatically omitted. Control variables are added incrementally: column (1) includes age, column (2) adds hukou status, column (3) incorporates years of schooling, and column (4) introduces employment status and income (in logarithmic form).

**Table 4.** Assortative mating: Influence of husband’s fertility preference on wife’s fertility preference (OLS).

Dependent variable	Wife’s ideal number of children			
Variables	(1)	(2)	(3)	(4)
Husband’s ideal	0.337 ***	0.329 ***	0.321 ***	0.320 ***
	(0.0338)	(0.0330)	(0.0328)	(0.0327)
Husband’s ideal × Sex ratio	0.0188	0.0178	0.0184	0.0180
	(0.0214)	(0.0210)	(0.0208)	(0.0207)
Husband’s Age	Yes	Yes	Yes	Yes
Husband’s Hukou	No	Yes	Yes	Yes
Husband’s Education	No	No	Yes	Yes
Husband’s Employment	No	No	No	Yes
Province FE	Yes	Yes	Yes	Yes
Observations	2,351	2,351	2,351	2,351
R-squared	0.314	0.315	0.327	0.328

Note: The estimated coefficients of control variables are displayed in Table A2. Robust standard errors in parentheses.

*** p<0.01,

** p<0.05,

* p<0.1.

**Table 5.** Assortative mating: Influence of wife’s fertility preference on husband’s fertility preference (OLS).

Dependent variable	Husband’s ideal number of children			
Variables	(1)	(2)	(3)	(4)
Wife’s ideal	0.511 ***	0.503 ***	0.497 ***	0.491 ***
	(0.0327)	(0.0332)	(0.0325)	(0.0323)
Wife’s ideal × Sex ratio	0.0432 **	0.0437 **	0.0431 **	0.0436 **
	(0.0183)	(0.0182)	(0.0183)	(0.0181)
Wife’s Age	Yes	Yes	Yes	Yes
Wife’s Hukou	No	Yes	Yes	Yes
Wife’s Education	No	No	Yes	Yes
Wife’s Employment	No	No	No	Yes
Province FE	Yes	Yes	Yes	Yes
Observations	2,351	2,351	2,351	2,351
R-squared	0.278	0.280	0.281	0.283

Note: The estimated coefficients of control variables are displayed in Table A3. Robust standard errors in parentheses.

*** p<0.01,

** p<0.05,

* p<0.1.

We use the scenario that includes all demographic characteristics as the benchmark, as shown in column (4) of both tables. The findings reveal that both husbands and wives exhibit a positive association between their own preferences for more children and those of their partners. All estimates are highly significant at the 1% level. Specifically, husbands whose ideal number of children exceeds the average male preference by one tend to have wives whose preferences are 0.32 higher than the average female preference. Similarly, wives whose ideal number exceeds the average female preference by one are associated with husbands whose preferences are 0.49 higher than the male average.

Males’ and females’ correlation to their spouse’s preference exhibit asymmetry when considering the provincial-level sex ratio. We use the sex ratio for those aged 20-39 in a province to capture the potential population in the marriage market. A larger sex ratio, indicating more males than females, serves as a proxy for women’s higher bargaining power. This is because a higher sex ratio intensifies competition among males for potential female partners, thereby enhancing women’s bargaining position in the marriage market and in family outcomes. In provinces where the sex ratio is one percentage point higher than the national average, the husband’s preference is much more strongly associated with the wife’s preference. More specifically, wives who prefer one more child than the average female ideal number tend to have husbands who prefer 0.53 (0.491 + 0.0436) more children than the average male ideal number. Conversely, the husband’s fertility preference is associated with his wife’s preference, but this association does not significantly vary with the sex ratio in their province of residence. This suggests that greater bargaining power—driven by women’s smaller numbers in the marriage market—enables them to pair with partners whose fertility preferences align more closely with theirs.

For men, the estimation results indicate that men cannot show a stronger association with their partner’s preference, even in provinces with a smaller ratio of men, because there are more men than women in the Chinese marriage market to begin with.

### Heterogeneity analysis

To further explore how these relationships vary across different demographic groups, we conduct a heterogeneity analysis based on educational level and hukou type. We define individuals with education above high school as highly-educated, and otherwise as less-educated. Hukou type is divided into either rural or urban.

First, we observe the results on the relationship between both spouses’ fertility preferences and the realization of the second-child fertility outcome. Tables A4 and A5 present the heterogeneity analysis by educational levels and hukou types, respectively, where the unit of observation is the household, and the sample is divided based on both spouses’ characteristics.

Among couples in which the wife is highly educated (regardless of the husband’s education level), the second-child fertility outcome is associated with both spouses expressing a preference for a second child, whereas individual preferences alone do not show significant associations. Among couples in which the wife is less educated (regardless of the husband’s education level), the insignificant interaction terms imply a lack of evidence for complementarity. These results suggest that complementary effects are more pronounced among highly-educated women. The weighted (weighted by subgroup sample sizes) average treatment effect of the interaction term is 0.190, indicating that when one spouse prefers a second child, the marginal association between the other spouse’s preference and the realization of a second birth increases by 0.19.

Among urban–urban, rural–rural, and urban-husband/rural-wife couples, complementary fertility preferences are positively associated with fertility outcomes. In contrast, for urban-wife and rural-husband couples, the wife’s preference appears to play a dominant role in determining the fertility outcome. This suggests that when the wife holds a more advantageous social status (i.e., urban hukou) than the husband, her fertility preference alone is more likely to determine the fertility outcome. Averaging different effects in different subgroup, the weighted average treatment effect of the interaction term across those different groups is 0.196, indicating that when one spouse prefers a second child, the marginal association between the other spouse’s preference and the realization of a second birth increases by 0.196.

Next, we observe that the results vary across subgroups defined by educational level and hukou type. Tables A6 and A7 present the heterogeneity analysis on the association between a husband’s (or wife’s) fertility preference and that of his (or her) spouse. The sample is divided based on individuals’ educational level and hukou status.

Among men, husbands who are either highly-educated urban, highly-educated rural, or less-educated rural exhibit a stronger association with their wives’ fertility preferences. In contrast, for all subgroups of women, a consistently strong association is observed between their preferences and those of their husbands. Averaging those effects in different subgroups, the weighted average treatment effect across subgroups is 0.333 (0.470), indicating that a one-child increase in husband’s (wife’s) preference corresponds to 0.33 (0.47) more in wife’s (husband’s) preference.

Heterogeneity analysis provides more detailed information. Consistent with the baseline results, variation is only observed in how husbands’ fertility preferences are associated with their wives’ preferences, but not in the opposite direction. In addition, the effect differs across subgroups.

For less-educated urban women, a higher sex ratio is associated with a weaker alignment between their preferences and those of their husbands; while for less-educated rural women, it is associated with a stronger alignment. This suggests that in rural areas, when there are fewer females than males, couples tend to have similar fertility preferences. The weighted average effect of the interaction term is 0.0458, indicating that in provinces where the sex ratio is one percentage point higher, wives who prefer one more child than the female average tend to have husbands whose preferences are approximately 0.52 (0.4696 + 0.04578) children higher than the male average.

## Discussion

Our study examines how both spouses’ fertility preferences are associated with fertility outcomes and whether assortative mating is based on fertility preferences in the Chinese context. Our first results suggest that having the same fertility preferences has additional complementary effects on having a second child. Specifically, the results indicate that spouses’ preferences are complementary. When a husband (wife) desires a second child, the association between their own preference and the actual fertility outcome becomes more pronounced if their partner also expresses a desire for a second child. This effect is more pronounced in couples with a highly-educated wife or an urban-hukou husband. These findings align with fertility studies from a household bargaining perspective and are consistent with previous empirical findings in multiple countries, where both spouses’ fertility preferences influence actual fertility outcomes (e.g., Thomson (1997), Thomson and Hoem (1998), Testa et al. (2014), Hener (2015), and Osiewalska (2018)).

Our second results suggest the non-random marriage matching by fertility preference, as individuals are more likely to pair with partners who share the same preference. It is a novel perspective contributing to the literature, where previous literature mostly focuses on personality traits, such as age, weight, height, health, and risk attitudes (e.g., Choo and Siow, 2006; Legros and Newman, 2007; Dupuy and Galichon, 2014; Chiappori et al., 2024), or socioeconomic characteristics, such as education, skill, income, and social status (e.g., Abramitzky et al., 2011; Han et al., 2015; Eika et al., 2019; Persson, 2020).

Our findings—that men and women consider preferences regarding the desired number of children when selecting a partner—have significant policy implications. In China, despite the relaxation of the one-child policy, the birth rate has declined since 2016. Notably, the marriage rate began to decrease two years prior to this decline. This suggests that the decrease in birth rates was likely driven primarily by the decline in marriage rates, as illustrated in Figure A1.

A natural question arises: why did the marriage rate begin to decline during this period? Our findings provide a potential explanation. Before the relaxation of the one-child policy, couples had only two fertility options: to have no children or to have one child. With such limited choices, the likelihood of disagreement over the desired number of children when selecting a partner was relatively low. However, after the policy’s relaxation, couples were presented with more options regarding family size. This increased the likelihood of disagreement over fertility preferences, making it more difficult for men and women to find an ideal partner. This difficulty may have contributed to the decline in marriage rates. Ironically, if this is the case, it suggests that a policy designed to encourage higher birth rates may have inadvertently contributed to their decline by leading to a drop in marriage rates.

Third, our analysis demonstrates that females with greater bargaining power, proxied by the provincial-level sex ratio for the population aged 20–39, have a stronger positive correlation with their husbands’ fertility preferences regarding the desired number of children, whereas males do not show a similar pattern. This suggests that women’s increased bargaining power influences assortative mating owing to their smaller numbers in the marriage market, enabling them to match with partners whose fertility preferences align with theirs. This aligns with previous literature on the impact of the sex ratio on marriage matching (e.g., Abramitzky et al., 2011; Brainerd, 2017), while being complemented by the new identifier of fertility preferences.

Lastly, our study complements the literature on the positive outcomes of a skewed sex ratio, which is often viewed negatively because of cultural values favoring “son preference” and the resulting prevalence of “missing girls” ( Almond et al., 2019; Qian, 2008). Recent studies have highlighted the positive effects of the skewed sex ratio, such as increased education and greater human capital investments, which enhance women’s empowerment in the labor market ( Huang et al., 2021). Our study demonstrates another positive outcome: a skewed sex ratio can grant women greater bargaining power in partner selection, enabling them to choose or influence partners to align with their fertility preferences.

In this paper, we can decompose the association between individual fertility preferences and fertility outcomes into two components: assortative mating based on fertility preferences and the complementarity of shared preferences within couples, assuming that the estimated coefficients can be interpreted as the causal relationship instead of association. Table 6 presents the overall association between individual fertility preferences and the likelihood of having a second child, calculated through these two mechanisms. Details of the calculation are provided in Appendix B1.

**Table 6.** The overall association between individual fertility preferences and actual fertility outcomes.

Gender		Men	Women
Category		(1)	(2)
Panel A. Education Level			
	Highly Educated	0.3539	0.3867
	Less Educated	0.4715	0.4850
Panel B. Hukou Type			
	Urban	0.3250	0.3681
	Rural	0.4281	0.4715

Note: The results are calculated based on the coefficients from Tables A4, A5, A6, and A7. Detailed calculation steps are provided in Appendix B1.

Table 6 highlights a fundamental tension between efficiency and equity, a common dilemma in many areas of economic policymaking. On one hand, our results indicate that highly educated and urban individuals (both men and women) who express a desire for a second child often face barriers that prevent them from realizing this preference. These obstacles may include challenges in finding a suitable partner, coordinating dual careers, or navigating social expectations. From a needs-based perspective, government support for such groups could be justified to help them realize their reproductive goals. On the other hand, from the standpoint of economic efficiency, such support may yield limited results if these barriers are difficult to overcome. Our estimates suggest that fertility preferences among rural and less-educated individuals are more strongly associated with actual fertility behavior, implying that policy interventions targeted at these groups may have a larger aggregate effect on fertility outcomes.

However, prioritizing support based solely on efficiency raises important equity concerns. All individuals should have equal rights to realize their fertility preferences, and policies must respect reproductive autonomy—especially for women. Therefore, the design of optimal fertility-related policies must strike a careful balance between efficiency, equity, and autonomy. The appropriate policy choice ultimately depends on the government's objective function, but our findings suggest that such decisions must be guided by a holistic consideration of both outcomes and ethical principles.

## Conclusions

This study examines fertility outcomes regarding the second child by utilizing data from the 2018 China Family Panel Studies survey. Furthermore, it explores assortative mating based on fertility preferences, using the provincial-level sex ratio from 2020 for the population aged 20-39 as a proxy for women’s bargaining power, which captures the potential population in the marriage market. Both analyses were conducted using Ordinary Least Squares regression models.

Our findings, based on a sample of 2,351 household-level observations, show that spouses’ preferences for the second child are complementary. When a husband (wife) wants to have a second child, the marginal effect of such preference on having the second child becomes higher when the wife (husband) wants to have the second child. This effect is particularly evident among couples with a highly educated wife or those with a male-hukou advantage (i.e., the husband’s hukou status is at least equal to the wife’s). Additionally, our research indicates that marriage matching is not random—individuals are more likely to pair with partners who share the same preference for the ideal number of children. Further analysis reveals that females with greater bargaining power exhibit a positive correlation with their husbands’ ideal family size, whereas males do not exhibit a similar correlation.

This study mainly contributes to the literature on assortative mating based on fertility preferences and its correlation with female bargaining power. To the best of our knowledge, this has not been explored in the previous literature.

## Data availability

### Underlying data

We are not allowed to distribute the original data since the data was collected by Peking University and the agreement with Peking University does not allows us to distribute the data. However, we provide the link to obtain the data from Pekin University and we provide the code to replicate our results. The original dataset used in this study is available from the following URL: https://opendata.pku.edu.cn/dataverse/CFPS?language=en.

### Extended data

Supplementary files are available at Open Science Framework (OSF) ( Zhuang and Naito, 2024): https://doi.org/10.17605/OSF.IO/F5DPZ.

Zhuang and Naito (2024) on OSF contains the following data:

• data.do: Stata file for data cleaning
• tables.do: Stata file for figures and regression analysis
• Tables.xlsx: Excel file for all tables
• Supplemental_Information.pdf: Tables A1 to A7, Figure 1A, and detailed calculation steps for Table 6

The above data are available under the terms of the Creative Commons Zero “No rights reserved” data waiver (CC0 1.0 Public domain dedication).

## Software and code

All analyses were conducted using Stata version 16.

The code to replicate our results are available on the above OSF domain.

Researchers who want to replicate our results can obtain the data set by proposing a research proposal to Peking University through the above website.

The findings, interpretations, and conclusions expressed in this article are entirely those of the authors and should not be attributed in any manner to Peking University, to its affiliated organizations.

### Reporting guideline

Repository: STROBE checklist for ‘Marital Bargaining and Assortative Matching on Fertility Preference: Evidence based on Cross-sectional Data in China’ is available at https://doi.org/10.17605/OSF.IO/2Z8HY.

Ethics and consent: we obtained the approval to use this data on August 9, 2021 from Peking University. Since this data is in the public domain and it is widely used, the review thorough the ethical committee at the University of Tsukuba was not needed. The original data collection project by Peking University was approved by the Biomedical Ethics Committee of Peking University, Beijing, China. The ethical approval number is IRB00001052-14010. We obtained the permission to publish this research on June 2 ^nd^ 2024 from Peking University.

## Footnotes

^1^
The first phase of the selective two-child policy (STCPI) was announced in 2011, allowing couples who were both singletons to have two children. The second phase (STCPII), from 2013 to 2015, extended the standard to couples in which at least one of the marital partners was an only child. In October 2015, the government declared that all couples in China’s mainland could have two children, which marked the official end of the one-child policy.

^2^

Mazzocco (2007) applies the collective model within a dynamic framework to study household decisions, with (cooperative) and without commitment (non-cooperative) and finds that the cooperative model with full commitment is rejected by the U.S. data. Similar tests of commitment have also been conducted by Rasul (2008) and Doepke and Kindermann (2019).

^3^
Studies also examine fertility from men’s perspectives, particularly in patriarchal societies in the developing countries, where the husband often acts as the primary decision-maker within the household ( Tertilt, 2005).

^4^
Similar research on child quality by Rangel (2006) finds a positive effect of women’s increased bargaining power through a policy change granting cohabiting women the same alimony rights as formally married women in Brazil.

^5^
While extensive empirical work exists on fertility preference disagreement in other countries; for example, Thomson (1997) on the U.S., Thomson and Hoem (1998) on Sweden, Gipson and Hindin (2009) on Bangladesh, Testa et al. (2014) on Italy, Hener (2015) on German, and Osiewalska (2018) on 10 European countries.
