# Supplementary material for: Marital Bargaining and Assortative Mating on Fertility Preference : Evidence based on Cross-sectional Data in China
Source: F1000Res. 2025 Jul 30;13:659. Originally published 2024 Jun 19. [Version 4] doi: 10.12688/f1000research.151196.4 (PMC12351236; doi:10.12688/f1000research.151196.4)
Supplement: Peer review history

# Reviewer report: Reviewer response for version 4

**DOI:** 10.5256/f1000research.185696.r401597

**Published:** 2025-08-13

**Authors:** Stuart Gietel-Basten

**Affiliations:** 1 The Hong Kong University of Science and Technology, Hong Kong, SAR, China

No further comments! I recommend indexing this article.

Is the work clearly and accurately presented and does it cite the current literature?

No

If applicable, is the statistical analysis and its interpretation appropriate?

Yes

Are all the source data underlying the results available to ensure full reproducibility?

No

Is the study design appropriate and is the work technically sound?

Yes

Are the conclusions drawn adequately supported by the results?

Yes

Are sufficient details of methods and analysis provided to allow replication by others?

Yes

Reviewer Expertise:

Demography

I confirm that I have read this submission and believe that I have an appropriate level of expertise to confirm that it is of an acceptable scientific standard.

# Reviewer report: Reviewer response for version 3

**DOI:** 10.5256/f1000research.183278.r390975

**Published:** 2025-07-22

**Authors:** Stuart Gietel-Basten

**Affiliations:** 1 The Hong Kong University of Science and Technology, Hong Kong, SAR, China

I am grateful for the revisions which look really good. I would just like to make one very strong recommendation before fully approving. I think the policy recommendations need to be very careful in terms of the language used. Is it the role of government to be 'incentivising' childbearing? This really goes against the common best practice we see at the moment. Rather governments should be listening to the different needs and concerns that different groups have and responding to them. 

 Ultimately if the authors want to go with incentivising reproductive labour then I have no choice but to accept their view. However I would strongly advocate them to apply a policy recommendation more aligned with ICPD principles.

Is the work clearly and accurately presented and does it cite the current literature?

No

If applicable, is the statistical analysis and its interpretation appropriate?

Yes

Are all the source data underlying the results available to ensure full reproducibility?

No

Is the study design appropriate and is the work technically sound?

Yes

Are the conclusions drawn adequately supported by the results?

Yes

Are sufficient details of methods and analysis provided to allow replication by others?

Yes

Reviewer Expertise:

Demography

I confirm that I have read this submission and believe that I have an appropriate level of expertise to confirm that it is of an acceptable scientific standard, however I have significant reservations, as outlined above.

# Response

**Published:** 2025-07-25

**Authors:** Hisahiro Naito

**Affiliations:** University of Tsukuba, Japan

**Dear Professor Gietel-Basten,**

 Thank you very much for your constructive comment on our policy recommendation section. Following your suggestion, we have entirely rewritten this part to better reflect the importance of considering equity and individual autonomy in reproductive decision-making.

 Specifically, we now write:

 “Table 6 highlights a fundamental tension between efficiency and equity—a common dilemma in many areas of economic policymaking. On one hand, our results indicate that highly educated and urban individuals (both men and women) who express a desire for a second child often face barriers that prevent them from realizing this preference. These obstacles may include challenges in finding a suitable partner, coordinating dual careers, or navigating social expectations. From a needs-based perspective, government support for such groups could be justified to help them achieve their reproductive goals.

 On the other hand, from the standpoint of economic efficiency, such support may yield limited results if these barriers are difficult to overcome. Our estimates suggest that fertility preferences among rural and less-educated individuals are more strongly associated with actual fertility behavior, implying that policy interventions targeted at these groups may have a larger aggregate effect on fertility outcomes.

 However, prioritizing support solely based on efficiency raises important equity concerns. All individuals should have equal rights to realize their fertility preferences, and policies must respect reproductive autonomy—especially for women. Therefore, the design of optimal fertility-related policies must strike a careful balance between efficiency, equity, and autonomy. The appropriate policy choice ultimately depends on the government's objective function, but our findings suggest that such decisions must be guided by a holistic consideration of both outcomes and ethical principles.”

 We hope this revision aligns with your recommendation.

**Best wishes,**

# Reviewer report: Reviewer response for version 3

**DOI:** 10.5256/f1000research.183278.r390976

**Published:** 2025-06-24

**Authors:** Hanzhe Zhang, Jie Wu

**Affiliations:** 1 Economics, Michigan State University (Ringgold ID: 3078), East Lansing, Michigan, USA

Summary

 We appreciate the authors' thoughtful and careful responses to the previous review. The revised manuscript shows clear improvements, including a more appropriate use of causal language, more concrete policy discussions, and the addition of heterogeneity analyses. Minor issues such as typographical errors and figure labeling have also been addressed. Below, we offer two additional minor comments regarding the newly added section.

Minor Comments

1. In the third paragraph of the heterogeneity analysis section, the manuscript states that "individual preferences matter more" among couples in which the wife is less educated. However, this conclusion appears to be based primarily on the insignificance of the interaction term. We suggest clarifying this interpretation, as a non-significant interaction does not necessarily imply stronger individual effects. It may simply reflect a lack of evidence for complementarity. A more precise explanation would help avoid potential misinterpretation and strengthen the argument.

 

1. In the final paragraph of the heterogeneity analysis section, the paper states that "This suggests that in rural areas, when there are fewer females than males, women have greater bargaining power and choose husbands who have similar fertility preferences." This language may overstate what the data can support. Given the cross-sectional nature of the data, it is not possible to identify whether preference alignment results from premarital selection, post-marital adaptation, or unobserved confounding factors. While the association is consistent with assortative mating, a more cautious phrasing, such as "couples tend to have similar fertility preferences", would improve the credibility and precision of the interpretation.

Overall, the paper provides valuable insights into fertility preference alignment and assortative mating in China. The revised manuscript demonstrates meaningful improvements in addressing earlier concerns. With the additional refinements suggested above, the paper will be further strengthened.

Is the work clearly and accurately presented and does it cite the current literature?

Yes

If applicable, is the statistical analysis and its interpretation appropriate?

Yes

Are all the source data underlying the results available to ensure full reproducibility?

Yes

Is the study design appropriate and is the work technically sound?

Yes

Are the conclusions drawn adequately supported by the results?

Yes

Are sufficient details of methods and analysis provided to allow replication by others?

Yes

Reviewer Expertise:

marriage market

We confirm that we have read this submission and believe that we have an appropriate level of expertise to confirm that it is of an acceptable scientific standard.

# Reviewer report: Reviewer response for version 2

**DOI:** 10.5256/f1000research.177179.r381711

**Published:** 2025-05-27

**Authors:** Hanzhe Zhang, Jie Wu

**Affiliations:** 1 Economics, Michigan State University (Ringgold ID: 3078), East Lansing, Michigan, USA

Summary

 This paper examines the relationship between fertility decisions, specifically the decision to have a second child, and the fertility preferences of spouses in China. Using data from the 2018 China Family Panel Studies, the authors find that spouses’ preferences are complementary. Furthermore, the study explores how one partner’s fertility preference is associated with the others. The positive association between partners' ideal number of children provides empirical evidence of assortative mating based on fertility preferences.

 Importantly, the paper highlights an asymmetry in how male and female preferences respond to their partners’. By using provincial-level sex ratios as a proxy for women’s bargaining power in the marriage market, the authors show that in provinces where women have stronger bargaining power, wives’ preferences are more closely aligned with their husbands’ preferences. However, no similar pattern is observed for men.

 Overall, this paper offers valuable insights into assortative mating in the context of fertility preference, particularly within the Chinese institutional and demographic setting. It contributes to the literature by filling an important gap and introducing fertility preference as a new dimension for understanding partner matching dynamics.

Major Comments

1. While the paper presents interesting findings, it consistently uses causal language that may overstate the strength of the empirical design. For example, phrases like "how bargaining power affects assortative mating" (p5) and "how fertility preferences influence fertility decisions" (p12) suggest causality, despite the use of cross-sectional data without exogenous variation. This issue is particularly visible in Equation (1), where the outcome variable twochild which is defined as having two or more children reflects realized fertility, not the decision to have a second child. This measure may be endogenous to reported preferences, for example, families with more children are likely to report higher ideal family sizes, raising concerns about reverse causality. The authors should revise the language to reflect the correlational nature of the results, clarify the limitations of the outcome measure, and consider exploring quasi-experimental strategies in future work.  
2. The paper raises important questions with potential policy relevance, but the discussion of implications remains vague. Current policy suggestions such as preference disagreement reducing marriage rates are speculative and lack empirical support. Moreover, no actionable recommendations are provided. Clarifying the mechanisms and offering specific, evidence-based policy suggestions would enhance the contribution of the paper.
3. The paper would also benefit from analyzing heterogeneity across key subgroups. Although hukou, income, and education are included as controls, this assumes homogeneous effects. In reality, preference alignment may matter more in urban or highly educated couples, while rural or less-educated groups may be influenced by family structure or constraints. Subgroup analysis would uncover whether the observed patterns vary across social contexts, clarify which groups are driving fertility decline, and help inform more targeted and effective policy design.

Minor Comments

1. Typo: There is a repetition of “such as” on page 12. Please revise for clarity.
2. On page 12, in Figure 3, the caption mentions short-dash, dash, and long-dash lines indicating the starting times of different policies, but these are not clearly visible or differentiated in the figure. Please clarify or revise the figure accordingly.

Overall Evaluation

 This is a timely and well-motivated paper that addresses a relevant demographic issue with a novel focus on fertility preference alignment between spouses. With clearer causal framing, more specific policy interpretation, and exploration of heterogeneity, the paper has strong potential to make a meaningful contribution to the literature on fertility behavior and household decision-making in developing contexts. I encourage the authors to revise the manuscript considering the above comments.

Is the work clearly and accurately presented and does it cite the current literature?

Yes

If applicable, is the statistical analysis and its interpretation appropriate?

Yes

Are all the source data underlying the results available to ensure full reproducibility?

Yes

Is the study design appropriate and is the work technically sound?

Yes

Are the conclusions drawn adequately supported by the results?

Yes

Are sufficient details of methods and analysis provided to allow replication by others?

Yes

Reviewer Expertise:

marriage market

We confirm that we have read this submission and believe that we have an appropriate level of expertise to confirm that it is of an acceptable scientific standard, however we have significant reservations, as outlined above.

# Response

**Published:** 2025-06-03

**Authors:** Hisahiro Naito

**Affiliations:** University of Tsukuba, Japan

Dear Professor Hanzhe Zhang and Professor Jie Wu,

 We appreciate the constructive comments and suggestions you provided. Your comments helped us to imporove the quality and clarity of our manuscript significantly.

 We appreciate the reviewer’s constructive feedback. In response, we have made two major revisions in the revised draft. First, following the reviewer’s suggestion to explore subgroup differences, we added a heterogeneity analysis based on education level and hukou type at the end of the results section. This addition helps us better understand how the complementary effects and preference alignment vary across social contexts, and which groups contribute more to fertility outcomes.

 Second, in response to the reviewer’s comment regarding the vagueness of the policy discussion, we have expanded the discussion section to include clearer and more evidence-based policy implications. To avoid speculative statements, we have also moved Figure 3 to the appendix, as it is intended for reference only and is not supported by empirical analysis in this paper.

 We have commented below on each of the points raised by the referee:

**Comment 1:** While the paper presents interesting findings, it consistently uses causal language that may overstate the strength of the empirical design. For example, phrases like "how bargaining power affects assortative mating" (p5) and "how fertility preferences influence fertility decisions" (p12) suggest causality, despite the use of cross-sectional data without exogenous variation. This issue is particularly visible in Equation (1), where the outcome variable twochild which is defined as having two or more children reflects realized fertility, not the decision to have a second child. This measure may be endogenous to reported preferences, for example, families with more children are likely to report higher ideal family sizes, raising concerns about reverse causality. The authors should revise the language to reflect the correlational nature of the results, clarify the limitations of the outcome measure, and consider exploring quasi-experimental strategies in future work. 

**R** **eply:** We have revised the language throughout the paper to avoid implying causality. We replaced words like “affect,” “influence,” and “effect” with more neutral terms such as “is/are associated with.” To avoid the endogeneity concern that our cross-sectional dataset reflects realized fertility outcomes rather than fertility decisions, we replaced the term “fertility decision” with “fertility outcome” to more accurately reflect the nature of the data. As a clear example of this revision, we changed “…their own preference has a stronger influence on the actual decision to have a second child” to “…their own preference is more strongly associated with the realization of a second birth…” (6th paragraph in the Introduction section). These changes aim to more accurately reflect the correlational nature of our analysis. In addition, we have added a paragraph near the end of the Introduction section to acknowledge the limitation of not establishing causal effects and to suggest that future research could explore quasi-experimental strategies to address this issue.

**Comment 2: **The paper raises important questions with potential policy relevance, but the discussion of implications remains vague. Current policy suggestions such as preference disagreement reducing marriage rates are speculative and lack empirical support. Moreover, no actionable recommendations are provided. Clarifying the mechanisms and offering specific, evidence-based policy suggestions would enhance the contribution of the paper.

**Re** **ply:** In the revised draft, we have expanded the discussion of policy implications to provide more specific and evidence-based recommendations, as shown in the last two paragraphs of the discussion section. In particular, we calculate the marginal effect of individuals’ fertility preferences on fertility outcomes and discuss how it varies by education level and hukou type. We further suggest targeting financial incentives toward subgroups with larger marginal associations—such as less-educated and rural individuals—to improve policy efficiency. To better explain the different results across demographic and socioeconomic groups, we added Table 6 to show the marginal effects across subgroups by education level and hukou type, and in Appendix 2, we provide the detailed calculation steps for the marginal effects.

 We have also revised the speculative discussion regarding the relationship between preference disagreement and declining marriage rates. To avoid overinterpretation, we moved Figure 3 to the Appendix and clearly noted that it serves only as contextual background without empirical testing.

**Comment 3:** The paper would also benefit from analyzing heterogeneity across key subgroups. Although hukou, income, and education are included as controls, this assumes homogeneous effects. In reality, preference alignment may matter more in urban or highly educated couples, while rural or less-educated groups may be influenced by family structure or constraints. Subgroup analysis would uncover whether the observed patterns vary across social contexts, clarify which groups are driving fertility decline, and help inform more targeted and effective policy design.

**Re** **ply:** In the revised manuscript, we added a detailed heterogeneity analysis by education level and hukou status at the end of the Results section. To keep the main text concise, the full regression tables are included in the Appendix (Tables A4–A7). Tables A4 and A5 examine heterogeneity in second-child fertility outcomes by couples’ education and hukou type, while Tables A6 and A7 focus on assortative mating—how one partner’s fertility preference is associated with the other’s. The final columns in each table report the weighted average marginal effects, accounting for subgroup sizes.

 This analysis allows us to explore whether the complementary effects of spousal preferences and preference alignment vary across social groups. We find that couples with a highly educated wife or those with a male-hukou advantage exhibit stronger complementary effects on fertility outcomes. Furthermore, a higher sex ratio is associated with stronger preference alignment among less-educated rural women, but weaker alignment among their urban counterparts. These findings provide new insight into the social contexts in which fertility preferences translate into behavior and offer implications for more targeted and effective policy design.

**Comment 4:** Typo: There is a repetition of “such as” on page 12. Please revise for clarity.

**Re** **ply:** Thank you for pointing this out. We have removed the redundant phrase for clarity.

**Comment 5:** On page 12, in Figure 3, the caption mentions short-dash, dash, and long-dash lines indicating the starting times of different policies, but these are not clearly visible or differentiated in the figure. Please clarify or revise the figure accordingly.

**Re** **ply** **:** We revised Figure 3 to clearly differentiate the short-dash, dash, and long-dash lines. Additionally, we moved the figure to the appendix, as it provides contextual information rather than empirical evidence and we wish to keep the main text focused on the core analysis.

 Thank you again for your valuable feedback. We hope the revised manuscript meets the standards of the journal.

 Meiyi Zhuang

 Graduate School of Humanities and Social Sciences, University of Tsukuba

 Email: z.meiyi95@gmail.com

 Address: Graduate School of Humanities and Social Sciences, 1-1-1, Tennodai, Tsukuba City, Ibaraki Prefecture, Japan Postal Code: 305-8573.

 Hisahiro Naito

 Corresponding Author

 Graduate School of Humanities and Social Sciences, University of Tsukuba

 Email: naito@dpipe.tsukuba.ac.jp

 Address: Graduate School of Humanities and Social Sciences, University of Tsukuba, 1-1-1, Tennodai, Tsukuba City, Ibaraki Prefecture, Japan Postal Code: 305-8573.

# Reviewer report: Reviewer response for version 1

**DOI:** 10.5256/f1000research.165824.r294203

**Published:** 2024-08-20

**Authors:** Stuart Gietel-Basten

**Affiliations:** 1 The Hong Kong University of Science and Technology, Hong Kong, SAR, China

A good paper using an excellent data set. The analysis is simple but suffices. Some issues:

- The literature review is somewhat haphazard and could be made more systematic and comprehensive.
- The conclusion section is far too short. There should be a discussion section which, well, discusses the findings, expanding on limitations, policy implications and so on.
- The terminology throughout should be more careful (e.g. 'birth rate')
- The first figure doesn't make sense (has no y axis label, doesn't seem to match the description etc).

In short the paper is ok, but the authors need to tighten it up a lot and be much clearer and specific about its contribution to the literature (which includes a more expansive literature review to point out the gaps).

Is the work clearly and accurately presented and does it cite the current literature?

No

If applicable, is the statistical analysis and its interpretation appropriate?

Yes

Are all the source data underlying the results available to ensure full reproducibility?

No

Is the study design appropriate and is the work technically sound?

Yes

Are the conclusions drawn adequately supported by the results?

Yes

Are sufficient details of methods and analysis provided to allow replication by others?

Yes

Reviewer Expertise:

Demography

I confirm that I have read this submission and believe that I have an appropriate level of expertise to confirm that it is of an acceptable scientific standard, however I have significant reservations, as outlined above.

# Response

**Published:** 2025-01-18

**Authors:** Hisahiro Naito

**Affiliations:** University of Tsukuba, Japan

Dear Professor Stuart Gietel-Basten,

 Thank you for your thoughtful comments and suggestions. They have been instrumental in improving our paper. We have made two major revisions in response to your feedback:

**1.    Expanded the Literature Review and Discussion sections (Sections 2 and 5)**

 We have added a more comprehensive literature review (Section 2) to provide a systematic overview of current studies on fertility decisions (Section 2.1), assortative mating (Section 2.2), and their application in the Chinese context (Section 2.3). In these sections, we explicitly identify the research gap (page 8, fourth paragraph) and highlight our paper’s contribution (page 8, fifth paragraph). Additionally, we have expanded our discussion to address empirical findings, policy implications, and limitations (Section 5, “Discussion,” pages 16–18).

**2.    Refined Empirical Analysis**

 We now include households in which spouses originate from different provinces, increasing our sample size from 2,054 to 2,351. Consequently, the parameter estimates in all five tables have been slightly revised. We have also incorporated province fixed effects into equations (1)–(3) to account for province-specific factors such as culture and traditions. Owing to these fixed effects, the estimated coefficient of the provincial-level sex ratio is omitted.

 Additionally, we slightly modified the title to reflect the revisions. The previous title was “Marital Bargaining and Assortative Matching on Fertility Preference : Evidence based on Cross-sectional Data in China.” The current revised title is “Marital Bargaining and Assortative Mating on Fertility Preference : Evidence based on Cross-sectional Data in China.”  For the revision, please find our responses to each of your comments:

**Comment 1: **“The literature review is somewhat haphazard and could be made more systematic and comprehensive.”

**Reply: **Thank you for the suggestion. We have introduced Section 2, Literature Review (pages 6–8), which is divided into three parts: empirical studies on fertility decisions (Section 2.1), empirical studies on assortative mating (Section 2.2), and applications in the Chinese context (Section 2.3). In Section 2.3 (page 8, fourth paragraph), we explicitly outline the gap in the literature, and in the fifth paragraph, we discuss our contribution. These revisions offer a lucid, structured overview of existing research and situate our study within the broader academic landscape.

**Comment 2: **“The conclusion section is far too short. There should be a discussion section which, well, discusses the findings, expanding on limitations, policy implications and so on.”

**Reply: **Thank you for your helpful comments. In response, we have added Section 5, Discussion (pages 16–18). In this section, we first discuss our empirical findings (page 17, first two paragraphs), and thereafter, elaborate on their policy implications. Specifically, we suggest that the relaxation of the one-child policy—intended to raise the birth rate—may have inadvertently contributed to its decline by increasing disagreements over fertility preferences, complicating partner matching, and lowering marriage rates. To support this argument, we include an analysis of Chinese marriage and birth rate trends and illustrate the time gap between their respective declines in Figure 3 (page 17). We conclude this section by linking our findings to the existing literature, highlighting both the similarities in fertility decisions and the novel insights on assortative mating. We note that the study’s limitations are presented in the Introduction (page 5, second-to-last paragraph).

**Comment 3: **“The terminology throughout should be more careful (e.g. ‘birth rate’).”

**Reply:** Thank you for pointing out this issue. We have carefully reviewed the entire manuscript to ensure consistent use of terminology. Specifically, we have replaced “fertility rate” with “birth rate” wherever applicable.

**Comment 4: **“The first figure doesn’t make sense (has no y-axis label, doesn’t seem to match the description etc.).”

**Reply: **Thank you for drawing our attention to this oversight. We discovered that Figures 1 and 2 were mistakenly swapped in the previous version. We have corrected this error and added a y-axis label to the updated Figure 2:

 Figure 2: Distribution of Female Age in the Sample, N=2,351N = 2{,}351

 This figure now aligns properly with the accompanying description.

**Comment 5: **“In short the paper is ok, but the authors need to tighten it up a lot and be much clearer and specific about its contribution to the literature (which includes a more expansive literature review to point out the gaps).”

**Reply: **Thank you very much for your insightful comments. We have significantly expanded our literature review in Section 2, providing a detailed account of current studies on fertility and assortative mating. We highlight key gaps in multiple sections: the fifth paragraph of Section 1 (page 5), Section 2.3 of the literature review (page 8, fourth paragraph), and the final paragraph of Section 6 (Conclusion, page 19). For instance, we explicitly note that, to the best of our knowledge, no study has as yet examined the impact of fertility preferences on assortative mating. Furthermore, we explicitly articulate our contributions in the third paragraph of Section 1 (Introduction, page 5), Section 2.3 (Literature Review), and again in the last paragraph of Section 6 (Conclusion, page 19). We explain that our work contributes to the literature by examining how fertility preferences affect assortative mating and by exploring the correlation between assortative mating and female bargaining power.

 Thank you again for your valuable feedback. We trust that these revisions have made our manuscript more cohesive, rigorous, and lucid. We look forward to any further comments you may have.

 Sincerely,

 Hisahiro Naito

 Professor

 Graduate School of Humanities and Social Sciences

 University of Tsukuba

 On behalf of coauthor
